# Population genetic considerations for using biobanks as international resources in the pandemic era and beyond

**DOI:** 10.1186/s12864-021-07618-x

**Published:** 2021-05-17

**Authors:** Hannah Carress, Daniel John Lawson, Eran Elhaik

**Affiliations:** 1 Department of Animal and Plant Sciences, University of Sheffield, Sheffield, UK; 2 School of Mathematics and Integrative Epidemiology Unit, University of Bristol, Bristol, UK; 3 Department of Biology, Lund University, Lund, Sweden

**Keywords:** Bioinformatics, Population structure, Population stratification bias, Genomic medicine, Biobanks

## Abstract

The past years have seen the rise of genomic biobanks and mega-scale meta-analysis of genomic data, which promises to reveal the genetic underpinnings of health and disease. However, the over-representation of Europeans in genomic studies not only limits the global understanding of disease risk but also inhibits viable research into the genomic differences between carriers and patients. Whilst the community has agreed that more diverse samples are required, it is not enough to blindly increase diversity; the diversity must be quantified, compared and annotated to lead to insight. Genetic annotations from separate biobanks need to be comparable and computable and to operate without access to raw data due to privacy concerns. Comparability is key both for regular research and to allow international comparison in response to pandemics. Here, we evaluate the appropriateness of the most common genomic tools used to depict population structure in a standardized and comparable manner. The end goal is to reduce the effects of confounding and learn from genuine variation in genetic effects on phenotypes across populations, which will improve the value of biobanks (locally and internationally), increase the accuracy of association analyses and inform developmental efforts.

**Supplementary Information:**

The online version contains supplementary material available at 10.1186/s12864-021-07618-x.

## Background

Association studies aim to detect whether genetic variants found in different individuals are associated with a trait or disease of interest, by comparing the DNA of individuals that vary in relation to the phenotypes [1]. For example, the major-histocompatibility-complex antigen loci are the prototypical candidates that modulate the genetic susceptibility to infectious diseases. As a result, association studies aim to identify which loci may provide valuable information for strategising prevention, treatment, vaccination and clinical approaches [2]. Such cardinal questions striking the core differences between individuals, families, communities and populations, necessitated genomic biobanks.

The completion of the human genome allowed genomic biobanks to be envisioned. The International HapMap Project, practically the first international biobank [3], facilitated the routine collection of data for genome-wide association studies (GWAS) [4]. GWAS to improve clarity soon after became the leading genetic tool for phenotype-genotype investigations. Over time, GWAS have been used to identify associations between thousands of variants for a wide variety of traits and diseases, with mixed results. GWAS drew much criticism concerning their validity, error rate, interpretation, application, biological causation [5] and replication [6]. Since much of this criticism was due to spurious associations yielded from small sample sizes with reduced power of association analyses, major efforts were taken to recruit tens of thousands of participants into studies where their biological data and prognosis were collected. These collections served as the basis for what is considered today as a (genomic) biobank [7].

Today, biobanks are known as massive scale datasets containing many hundreds of thousands of participants from specified populations. Biobanks have brought enormous power to association studies. Although it was unclear whether these new databases would deliver their most ambitious promises, the potential of biobanks in enabling personalised treatment was noted before the technology matured. It was initially expected that these databases would lead to the rapid discovery of a better genetic understanding of complex disorders, allowing for personalised treatments [8]. However, it is now clear that this expectation was exaggerated [8]. For example, a comprehensive review of the genomics of hypertension on its way to personalised medicine concluded that despite the wealth of identified genomic signals, actionable results are lacking [9]. No new drugs for the treatment of hypertension were approved for more than two decades. Moreover, the tailoring of therapy to each patient has not progressed beyond considering self-reported African ancestry and serum renin levels [9]. Another example is autism, the most extensively studied (40 years) and heavily funded ($2.4B in NIH funding over the past ten years [10]) mental disorder with nearly three dozen biobanks [11]. Despite these major efforts at understanding the disorder, there is still no single genetic test for autism, not to mention genetic treatment [12]. These gloomy reports of the state of knowledge in two of the most studied complex disorders, which typically harness massive biobanks, were not what the biobank enthusiasts envisioned at the beginning of the century [8].

**Fig. 1:**
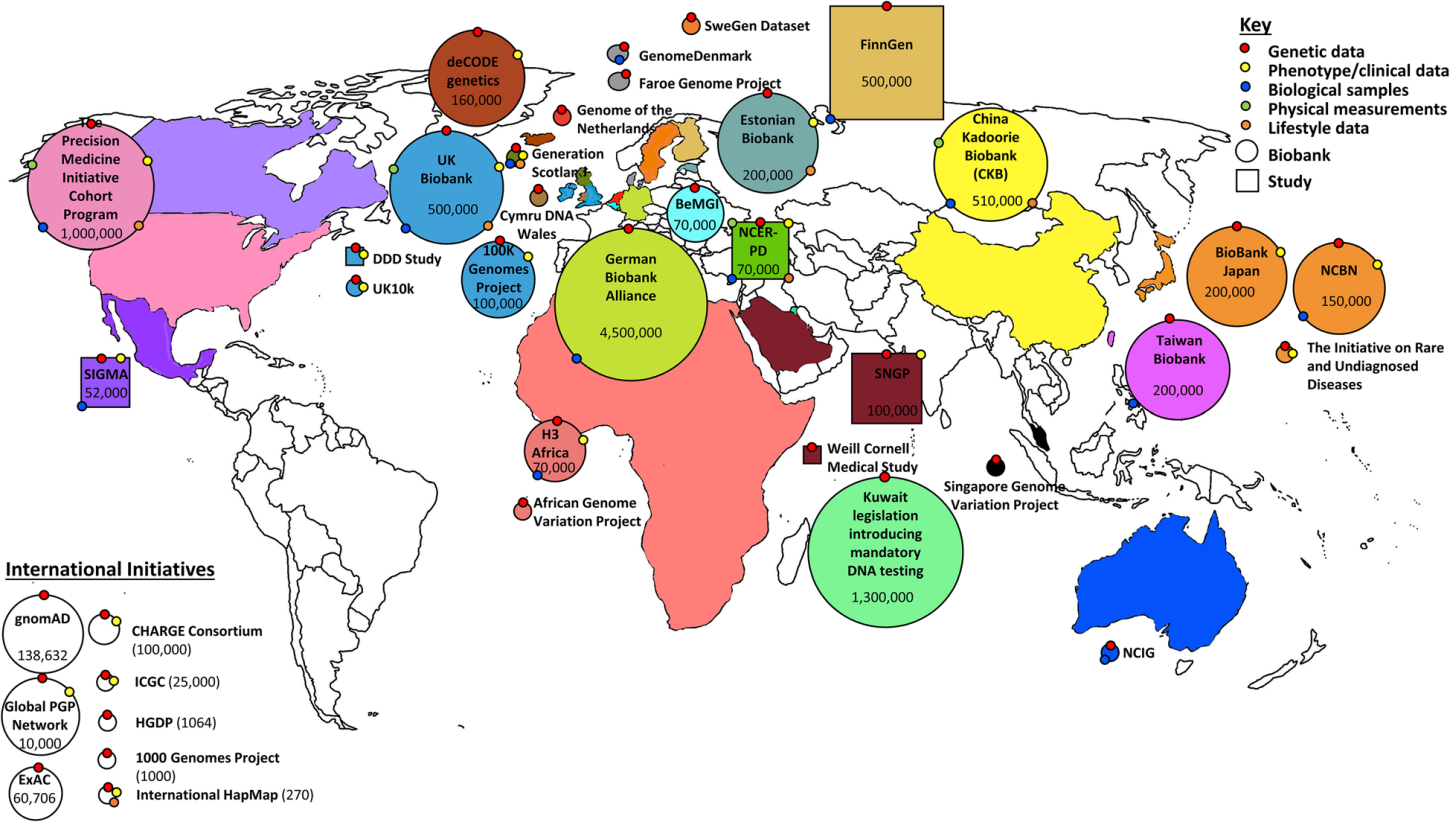
Global genomic biobanks (circles) and studies (squares). Databases vary by the type of data (see key) and their size. The map was created using R (v3.6) package ‘rworldmap’ (v1.3–6)

Back then, both private and government-sponsored banks began amassing tissues and data. For example, Generation Scotland [13] includes DNA, tissues and phenotypic information from nearly 30,000 Scots [14]; the 100,000 Genomes Project sequenced the genomes of over 100,000 NHS patients with rare diseases, aiming to understand the aetiology of their conditions from their genomic data [15]; and the UK Biobank project sequenced the complete genomes of over half a million individuals [16] with the aim of improving the prevention, diagnosis and treatment of a wide range of diseases [17]. Pending projects include the Genome Russia Project, which aims to fill the gap in the mapping of human populations by providing the whole-genome sequences of some 3000 people, from a variety of regions of Russia [18]. Biobanks are not without controversy. In Iceland, deCODE genetics has created the world’s most extensive and comprehensive population data collection on genealogy, genotypes and phenotypes of a single population. However, the economic value of the genomic data remained largely inaccessible, and the company filed for bankruptcy [19]. The experience of deCODE highlighted the risks in entrusting private companies to manage genomic databases, promoting similar efforts to have at least partial government control in the dozens of newly founded biobanks (reviewed in [20]), as illustrated in Fig. 1. Moreover, as the use of biobanks is expanding beyond their locality, for example, in the case of rare conditions where samples need to be pooled from multiple biobanks, the view of biobanks should be changed from locally-managed resources to more global resources. These should adhere to international standards to increase the accuracy of association studies and the use of biobanks [21].

Even past the formation of biobanks, many associations results failed to replicate (e.g., [22]) or show a difference in the effect across worldwide populations, in traits and disorders like body-mass index (BMI) [23], schizophrenia [24], hypertension [25] and Parkinsons’ disease [26]. Although strong associations between genetic variants and a phenotype typically replicated within the population that was studied, they may not have been replicated elsewhere. This leads naturally to further questioning the value and cost-effectiveness of association studies and biobanks [27] – what do the associations mean, and what are they useful for? How can we decide whether the association is relevant for different individuals, particularly those of mixed origins or those who may not know their origins? What are the considerations when designing a new biobank or merging data from multiple biobanks?

We argue that understanding population structure is a key component to answering these questions and contributing to the usefulness of biobanks and their ability to serve the general population [28–30]. In the following, we review the current state of knowledge on the importance of population structure to association studies and biobanks and the implications to downstream analyses. We then review biobank relevant models that describe population structure. We end with the challenges and benefits of the tools that implement these models.

## Main text

### Population diversity

Human genetic variation is a significant contributor to phenotypic variation among individuals and populations, with single-nucleotide polymorphisms (SNPs) being the most common form of genetic variation. Of the entire human genomic variation, only a paucity (12%) is between continental populations and even less genetic variation (1%) is between intra-continental populations [31]. In other words, a relatively small group of SNPs are geographically differentiated, whilst a much larger group of SNPs vary among individuals, irrespective of geography. However, most of these variants are rare and non-functional [32]. Both common and functional variants are strong predictors of geography, phenotypes and cultural practices that may be linked with the risk for a disease. Thereby, geographical and ancestral origins can not only inform us of what risk of disease an individual has, but also modify the effect of treatment [30]. In general, and with the clear exception for high admixture or migration followed by relative isolation [33–35], most associations between geographic location and genetic similarity are expected to hold worldwide (e.g., [36]). This is due to the exchange of genes and migrants between geographically proximate populations (e.g., [37–41]). These relationships are also expected to hold for common and rare variants [42]. The geographic differentiation between populations underlies their genetic variation or population structure, and studies in the field aim to analyse, describe or account for the genetic variation in time and space, within and among populations.

Unfortunately, worldwide diversity is widely misrepresented in GWAS studies [43]. By 2009, 96% of individuals represented in GWAS were of European descent [44]. This over-representation was rationalised by the interest to focus on ancestrally “homogenous” populations to avoid *population stratification bias*, i.e., systematic ancestry differences due to different allele frequencies in the studied cohorts that produced false positives [45]. Consequent efforts to carry out studies on non-Europeans were met with some success; by 2016, the proportion of Europeans included in GWAS declined to 81% [46] and further to 78% in 2019 [43]. However, even then, 71.8% of GWAS individuals are recruited from only three countries: the US, UK and Iceland [47].

**Fig. 2:**
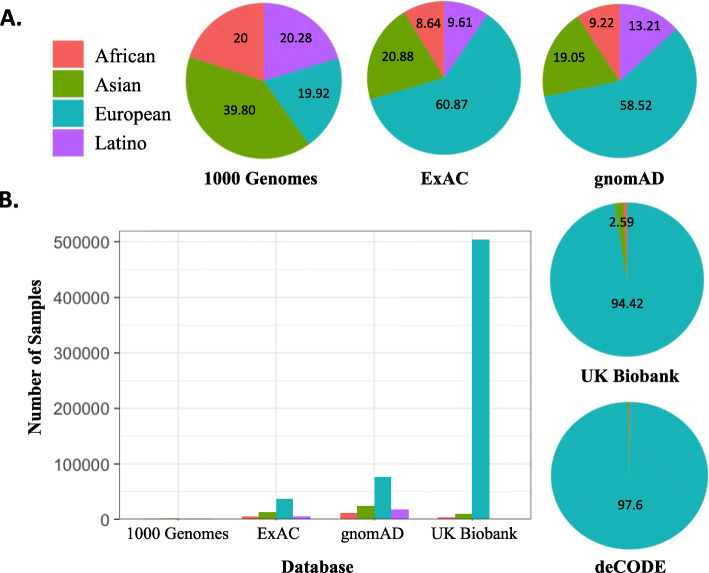
The **a** percentage and **b** number of samples in the 1000 Genomes Project, the ExAC browser, the UK Biobank and the gnomAD browser categorised into five ancestry groups: European, South Asian, African, East Asian and Latin (https://www.nature.com/articles/nature15393;http://exac.broadinstitute.org/faq;https://gnomad.broadinstitute.org/faq). The deCODE database has been circled in (**a**) and excluded in (**b**) because, when contacted, deCODE genetics were unable to disclose any information regarding the ancestry or number of samples; however, it can assumed that the database is roughly 97.6% European based on the finding of the recent consensus where 97.6% of the Icelandic population was defined as European (93% Icelandic and 3.1% Polish) [50]

Not all major genetic datasets are equally diverse, and most are skewed towards individuals of European ancestry (Fig. 2). For example, 61% of the samples in the Exome Aggregation Consortium (ExAC) dataset (60,252 individuals) [48], 59% of the Genome Aggregation Database (gnomAD) (141,456 individuals) [49], 94% of the UK Biobank database (500,000 individuals) [16] and an estimated 97.6% of the deCODE database are Europeans [50]. The UK Biobank was designed to be representative of the general population of the United Kingdom; however, that makeup is only 85% “White” [51]. Such misrepresentation of the global population structure has a detrimental impact on genomic medicine studies in England and international studies that rely on their results for several reasons: firstly, they promote a simplified view of “Europeans” as “homogeneous” [36]; secondly, ignorance of the global population structure prevents properly correcting the studies for *stratification bias*; and thirdly, the unequal representation of diversity within major genetic datasets increases the risk for false positives, due to chance or undetected population structure, and current methods to attempt to correct this underlying population structure are inadequate [23]. These limitations were highlighted during the COVID-19 pandemic, as the UK biobank data were shared internationally [52] to improve the response to the virus and protect the public represented in the biobank.

*Population stratification* may bias GWAS through two routes: the choice of the cohort and association analysis. Currently, individuals are matched and grouped mainly using self-reported “race” rather than genomic ancestry. This criterion is believed to account for the participants’ genetic background and supposedly allow controlling for population genetic structure (e.g., [53, 54]). A numerical example of how a false positive association can be created due to population stratification is demonstrated by Hellwege et al. [55].

However, grouping based on demographics alone does not account for differences in genetic ancestry between individuals, which leads to biased interpretation of the results or false negative or positive results [30, 56–59].

### Genomic medicine and diversity

*Personalised medicine* is thought of as the utilisation of epidemiological knowledge to produce a granular classification of patients into cohorts. These cohorts differ in their disease susceptibility, disease prognosis or response to treatment. It is considered the epitome of twenty-first century medicine [60]. To facilitate the accurate identification and classification of individuals into cohorts, it is necessary to consider their genomes, which lends credence to the development of *genomic medicine* and its aspired derivation, *personalised genomic medicine.*

*Genomic medicine* seeks to deploy the insights that the genetic revolution has brought about in medical practice [61]. The ability to predict individual risk of disease development, guide intervention and direct the treatment are the core principles of genomic medicine [62]. Most applications outside of simple Mendelian diseases start by considering known associations and testing for them in the sequence of the patient. Harnessing the knowledge gained from a small fraction of patients into the routine care of new patients has the potential to expand diagnoses outside of rare diseases, determine optimal drug therapy and effectiveness through targeted treatment, and allow for a more accurate prediction of an individual’s susceptibility to disease – the pillars of the genomic medicine vision [63].

**Fig. 3:**
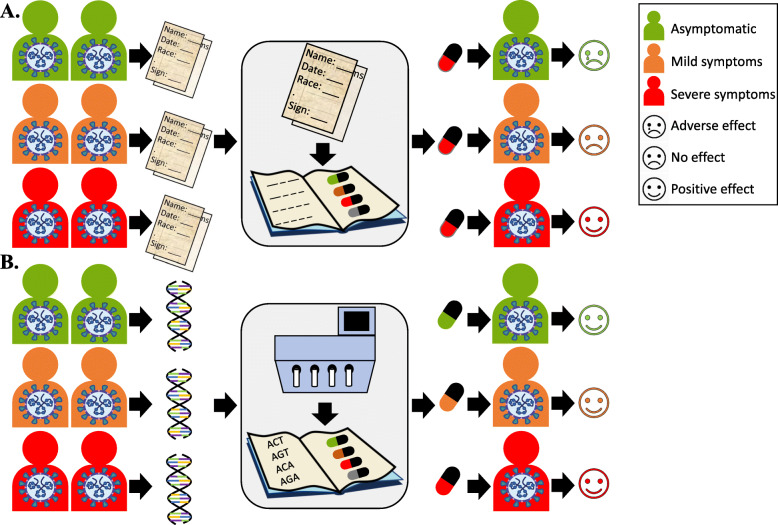
Using the example of COVID-19: **a** The current method of treatment whereby all patients with the same disease receive the same treatment. **b** Personalised medicine, whereby treatment is tailored to an individual to increase effectiveness

**Fig. 4:**
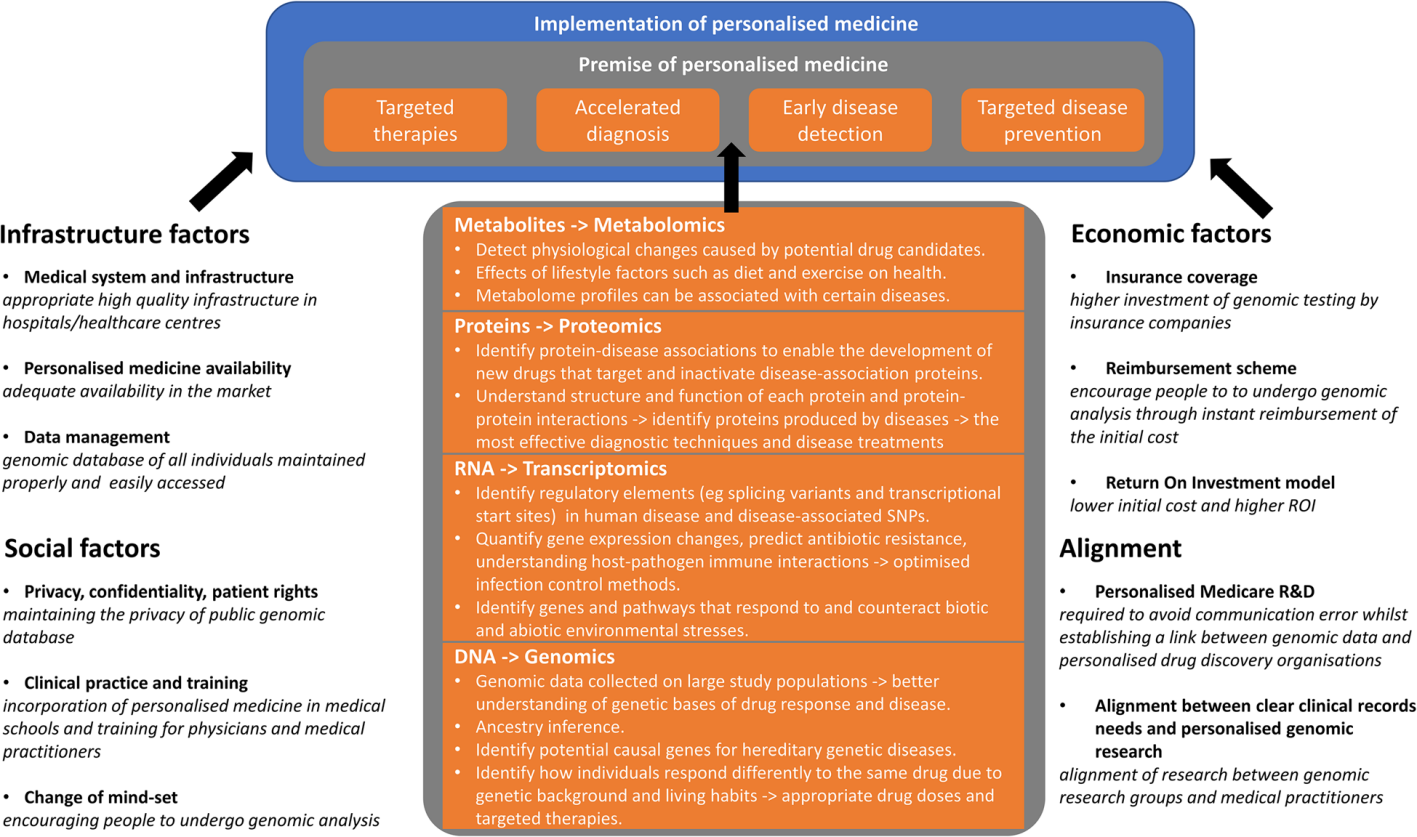
the road to personalised medicine. How the use of omics can be used to create the premise of personalised medicine (orange), which can be implemented into the healthcare system through the adoption of a variety of different factors (blue)

*Personalised genomic medicine* aims to tailor a treatment to an individuals’ genetic needs. This is expected to revolutionise disease treatment by using targeted therapy and treatment tailored to the individual to achieve the most effective outcome [64], as illustrated in Fig. 3. This form of genomic medicine was made feasible due to advances in computational biotechnology and its implementation into the health care system [65], illustrated in Fig. 4, alongside biological advancements that include the mapping of human genetic variation across the world through parallel global efforts [66]. However, it remains a futuristic vision rather than an everyday reality, due to the multiple obstacles that genetic studies face in deciphering complex genotype-phenotype relationships [67, 68]. One of the notorious difficulties in the field is the variation among population subgroups, which is often due to their genomic background [30]. Personalisation to the ancestral group-level is a more realistic short-term goal, yet being well-represented in genomic datasets is the exception rather than the rule. For example, an individual of Aramean ancestry living in the UK would be matched to only a handful of individuals in the UK Biobank. Similarly, individuals from Transcaucasia may be considered either “Europeans” or “Asians” and poorly represented by either, as their populations resemble an older admixture between these continental groups [36, 69]. The development of personalised medicine is, therefore, an area particularly affected by a lack of diversity in biobanks.

### Current biobank standards representing genetic variation

Accounting for population differences requires a reliable and global population structure model. Regrettably, despite the vast amount of genetic data currently available, no unified population structure model has been developed. Instead, population genetic studies typically describe variation in the data they study, sometimes with respect to related populations defined in a rudimentary way, for example, using the 14 (or even just the original four) HapMap populations [70] or 26 of the 1000 Genomes populations [42]. Unsurprisingly, without a model, correcting for population stratification remains strenuous.

Many association studies ignore population stratification or implicitly assume its redundancy if the data were collected from continental groups (e.g., [71]). Groups are assigned either by self-identified ancestry or inferred by comparison to the HapMap or 1000 Genomes populations, and each cluster is analysed independently (e.g., [71]). This approach does not account for the existence of fine-scale structure [23] and cannot be applied to more admixed populations, which is important where recent massive migrations have occurred, such as in the Americas.

### PCs and GRMs

Currently, “global correction” of such populations using either Principal Components Analysis (PCA see Supplementary Text S1, e.g., [72]) and/or mixed linear models (MLM, Supplementary Text S1, e.g. [73]) start with the Genetic Relatedness Matrix (GRM, Supplementary Text S1) [74] as the de-facto standard used to describe ancestry of large-scale genetic datasets. PCA aims to correct for the largest variation components of the GRM, whilst MLM aims to correct for the whole matrix, accounting for recently related individuals.

These tools view the genome as a set of independent loci whose effect can be simply added up. Unfortunately, depending on sampling and genetic drift, this can yield spurious results [58, 75–77] including representing individuals with two ancestrally different parents as similar to populations that resemble this mixture. For example, an individual with one European and one Asian parent may be incorrectly labelled as a Middle Eastern individual [58].

Both PCA and MLMs are used for meta-analyses of a large number of independent studies (e.g., BMI [78]). Meta-analysis demonstrates replication of effects of genetic risk loci and hence minimises individual cohort bias. However, the effect size estimate of meta-analysis is the averaged effect of the SNP on outcomes across several populations. The assumption that the effects of an SNP are equal across populations with different allele frequencies is unlikely to hold for three main reasons. Firstly, many SNPs identified in GWAS are not causal variants, but rather are in linkage disequilibrium (LD) with one or more causal variants, and LD patterns differ between populations [79]. Secondly, gene-environment interactions [80] may contribute to the overall effect of an SNP and these may differ by population (for example, in BMI and exercise, [81]). Thirdly, statistical artifacts can arise from differential correction power for stratification across studies [23]. The resulting bias is problematic because many downstream applications use summary statistics from GWAS and do not access the original dataset.

## Implications of population structure

Detecting associations between genotypes and phenotypes is only the beginning of the process. Different applications are, to various degrees, affected by a bias in the estimates of an effect, which is typically subjected to the very large variance for all but the strongest associations.

### Causal analysis using Mendelian randomisation

First outlined by Katan [82] and further developed by Davey-Smith and Ebrahim, [83], Mendelian Randomisation (MR) is a statistical approach in which genetic variants associated with an exposure of interest are used to examine the causal effect of said exposure on the disease. Because genotype is assigned at conception and common genetic variants are typically not associated with other lifestyle factors, these variants can be used as “instruments” for causal inference, limiting the problems of confounding and reversing causality that otherwise plagues observational epidemiology. MR may, therefore, offer an affordable and faster alternative to traditional RCTs [84, 85]. However, MR assumes that there is no confounding between the genetic polymorphism (which is a proxy for the exposure) and the disease outcome. If population stratification occurs due to mismatched ancestries, then this assumption will be violated, and any estimates will be biased. For instance, common genetic polymorphism in the CHRNA5-A3-B4 gene cluster that is related to nicotine dependence is often used as an instrument for tobacco smoke exposure. Assume that two alleles, *A* and *C*, exist at this polymorphic site, with those carrying the *A* allele exhibiting a tendency to smoke more cigarettes. Europeans without cryptic African/East Asian ancestry are unlikely to have the *A* allele regardless of their smoking practices, which may bias the MR study if ancestry is not properly accounted for in the study design. Within single studies where researchers have access to individual-level data, ancestry may be accounted for, to some extent, by adjusting for principal components. However, MR requires very large sample sizes, which necessitates collaboration across studies and meta-analysis, which may introduce genetic heterogeneity. MR’s susceptibility to population stratification is a well-recognised bias [86, 87] in case-control pharmacogenetics studies where differences in ancestry affect the results (e.g., weekly warfarin dose required to maintain a therapeutic effect varies by ancestry, likely due to genetic variation). Other MR limitations include a reliance on large GWAS, horizontal pleiotropy, and canalisation [88].

Two-sample Mendelian Randomisation (MR), in which the SNP-exposure association is estimated in one study and the SNP-outcome association is estimated in another, is important because it allows sharable summary statistics to be used for causal inference. Often one or both associations are determined using summary statistics and the researcher does not access the primary data [89]. Importantly, summary statistics are usually meta-analysed to determine an “average” SNP-exposure estimate across studies, and similarly, further studies are meta-analysed to determine the SNP-outcome estimate. Whilst in one step MR, there is an assumption that the effect of the SNP on the outcome and the effect of the SNP on the exposure is uniform across the populations included in any meta-analyses, two-sample MR makes a further assumption that the population in which the SNP-exposure estimate is determined is representative of the population in which the SNP-outcome association is determined (or that any differences are negligible). This assumption is questionable when combining an exposure GWAS from Han Chinese and an outcome GWAS from a Caucasian population, from which MR may produce biased results [90, 91]. Even the induced bias of using two different Caucasian populations (e.g., an exposure GWAS in a Scandinavian population and an outcome measured in a southern England population) is largely unknown. That bias would be most severe for rare conditions and small cohorts that include diverse individuals.

Recently, MR studies using a two-sample approach [92] have been automated using online platforms [93]. In an analysis that is limited to summary data (e.g., [71]), population stratification bias is difficult to identify, and the analysis is often run without adjustment for possible population differences. Sometimes the homogeneity of the dataset is assumed due to the continental affiliation of the cohort (e.g., [71, 94] analysed third-party summary statistics calculated for “Europeans”). LD score regression [95] can estimate the sample overlap between summary statistics, but this is reliant on relatively large samples and often not used in MR pipelines. MR assumptions and their consequent estimates would undoubtedly be more trustworthy if the underlying GWAS estimates were more universal and less population specific.

### Polygenic scores

Similar concerns were raised by multiple groups concerning polygenic scores. Sohail et al. [96] reported that polygenic adaptation signals based on large numbers of SNPs below genome-wide significance were found to be extremely sensitive to bias due to uncorrected population stratification. Berg et al. [97] analysed the UK Biobank and showed that previously reported signals of selection were strongly attenuated or absent and were due to population stratification. Both papers found that methods for correcting for population stratification in GWAS were not always sufficient for polygenic trait analyses and doubted the strength of the conclusions based on polygenic. Both papers, therefore, advised caution in their interpretation. Further concerns about polygenetic scores were raised by other groups [98–100].

### Drug discovery

GWAS are also used to identify druggable target genes [101]. Whilst it is not essential that the effect sizes are large, they must be associated with an underlying biological pathway [102]. There may be several reasons that limit the utilities of biobanks to identify drug targets, i.e., an association between a trait and genomic variant, like differences in lifestyle between populations, genetic interactions and genetic linkage. Since genetic variation is partly geographically differentiated, the frequencies of certain disease-causing genetic variants and variants in drug-metabolising genes may differ based on geographic location, leading to geographic disparities in the susceptibility of an individual to a disease and/or specific drug treatment [103–106]. As a result, the power to detect these unintended associations with a trait of interest is expected to grow with biobank size; therefore, correcting for population stratification will aid in the reduction of false-positive drug target leads. For pharmacogenetics to propel the practice of individualised drug therapy to become the standard of care [107], accurate genetic profiles should be constructed [30, 108] and genetic tools must be developed and verified toaccount for confounding effects using DNA sequencing analyses.

## Models for population structure

There are two cases to consider for modelling population structure: when the individual data for all populations are available and when they are not. With access to the individual data, a wide range of options exist, which can be broadly split again to within-dataset and cross-dataset analysis. Within-dataset analysis for biobanks must scale to hundreds of thousands of samples though need not naturally be comparable. Cross-dataset analyses would typically reference standard datasets, creating a comparable statistic for each individual. Depending on the usage, these references may not themselves be biobank scales. Meta-analysis using summary statistics resembles a cross-dataset analysis, with the further requirement of the creation of sharable summary statistics that remains meaningful without individual-level data.

This section summarises the current state of these methods, whilst the Usage section describes the challenges and benefits of the various tools that are available for each function.

### Describing genetic variation within a single dataset

#### Markers for ancestry

Genomic ancestry inference may employ specialized markers, such as ancestry informative markers (AIMs), which have significant differences in allele frequencies between populations. For instance, the T allele of the SNP rs316598 is very rare in Africans (3.3%) but common elsewhere and can, thereby, be used to differentiate Africans from non-Africans. AIMs, combined with other methods, can thereby be used to identify the origins of samples, provided that the genomes of worldwide reference populations are available [36, 58, 109].

One key advantage in using such markers to intensify the ancestry information, which can lead to its identification using downstream tools, is that frequencies of a particular dataset are sometimes already available as summary statistics. If frequency information has been released, then useful ancestry summaries can be extracted. However, to perform such an analysis in practice requires a global model to combine data together and form a meaningful and comparable report on ancestry for each dataset. This will typically require an examination of the methods to follow.

#### Low dimensional representations

PCA aims to reduce the dimensionality of the SNP dataset by reducing the genetic markers into principal components (PCs) [72, 110]. For population genetic inference, the results are hard to interpret, as the PCs do not mean anything intrinsically and often require more than two dimensions to correctly visualise [75]. Importantly, population structure is not always in the top PCs, especially under uneven sampling or genetic drift [58, 76]. However, it is fast to compute, which contributed to the popularity of PCA as a method of first choice. The results of PCA strongly depend on the choice of markers and samples, and interpretation is subjective without an actual measure of “close to” or “cluster with.” Since it has been suggested that PCs portray some geographic similarity within Europe [111], modified methods have been proposed, albeit with limited success. The* Spatial admixture analysis (SPA) *[112], for example, had a biogeographical prediction accuracy of 2% at the country level [36]. These methods cannot be readily applied to biobank-style data as they don’t scale [75].

Unfortunately, PCA results may not be reliable, robust, or replicable as the field assumes [75]. Given enough samples having been carefully chosen, it may be tweaked to form patterns that exhibit similarity to geography (e.g., over 100 K carefully chosen samples identified several broad regions in the UK (Fig. 1 in [113]). There are alternative and complementary approaches that require consideration.

#### Identity-by-state (IBS) and the Genetic relatedness matrix (GRM)

Identity-by-state (IBS) is often used to represent population structure and further represents relatedness (see below). IBS is the proportion of SNPs that are shared between each pair of individuals and therefore forms an *N* by *N* matrix of genetic similarity. Similarly, the association literature uses the “Genetic Relatedness Matrix” (GRM) [74], in which SNPs are centred by their frequency and weighted by their variance. The GRM is an important tool in mixed linear models that jointly address population structure and relatedness, perhaps the most common tools being *GCTA* [114] and *GTAK* [115]. The GRM can be shown to contain the same information as used by both admixture models and PCA (e.g., [116] supplementary material). The advantage of correcting for the complete matrix (rather than the low-rank approximation used in PCA) is that it retains the relatedness information. Otherwise, these procedures are asymptotically equivalent. Fast implementations exist, such as *Bolt-LMM* [117], but these may implicitly demonstrate the low-rank structure and hence lower correction power. Implementations like *LMM-OPS* [118] attempt to correct increased type-I error rates and a loss of power due to heterogeneous ancestry.

#### Ancestry as a mixture

Admixture or admixture-like analyses originated in the popular program *STRUCTURE* [119, 120]. Here, the ancestry of each individual is modelled as a proportion of *K* admixture components, which are learned automatically, and represent “historical populations” in the model. Whilst computation was historically a concern, fast enough implementations now exist (e.g., *faststructure* [121] and *terrastructure* [122]). However, the ancestral interpretation is often misleading [123], since sampling and genetic drift can also create the same representation for different true histories. Further, the choice of “proper” *K* is unclear [123, 124] and can have significant effects on the inference. Conceptually, ADMIXTURE uses the same information as PCA [116] and hence suffers from the same limitations [75].

#### Sibship, kinship and clanship via identity by descent

Since related individuals do not represent statistically independent samples and may lead to false-positive associations, association analyses containing related individuals require special care [125, 126]. The degree of relatedness is different between individuals and ranges between the largely known sibship, the often-known kinship, and the typically unknown clanship. Relatedness can be identified from DNA, as it is inherited in segments from one’s ancestors, whereby long segments are shared between more recently related individuals. This inheritance pattern is used to define genetic regions for two pairs of individuals that are identical by descent (IBD).

The difficulty in estimating relatedness increases as the relationship becomes more distant because the IBD DNA segments between individuals are shorter and more difficult to distinguish from DNA segments that are IBS. Typically, all the IBD segments that are more recent than a chosen (average) age of the pairwise relationship are sought by thresholding the lengths of segments.

Alone, IBD is not a measure of ancestry, though its results can be summarised into a Kinship matrix analogously to the GRM. Kinship is the probability that two homologous alleles drawn from each of two individuals are IBD [127, 128]. The value of IBD for detecting associations in biobanks has not been explored, likely due to the complexity of the calculation (*N* by *N* analyses), which is time-consuming. One possibility is to create an unbiased random sample of genes and traits by sampling only one version of each IBD tract since the two copies are clearly dependent. Other possibilities are to treat long IBD as a sparse property, reducing the need to generate a full pairwise matrix.

#### Haplotypes

IBD matches may overlap and may ignore some parts of the genome entirely. An alternative approach is to identify the closest relative for every individual at each position on the genome. This is the approach taken in Chromosome Painting [116], which allows the identification of fine-scale population structure beyond the detection limit of related approaches [129]. Chromosome Painting is applicable for samples up to thousands but cannot be used at biobank scale [130] because of the same problem of producing an *N* by *N* matrix. Considering large matrices of pairwise haplotype information (throughout the genome) is not trivial and remains a challenge for biobanks.

#### Local ancestry

The purpose of Local Ancestry Inference (LAI) is to analyse individual segments of DNA to establish changes in ancestral origin. Being able to assign an SNP as having originated in a particular ancestry, association testing can, in principle, be carried out in each ancestry as if it were a single sample population.

Conceptually, such methods examine a stretch of DNA and use a model related to the mixture approaches to identify the source population. The approaches vary in how appropriate stretches of DNA are defined and how they are matched to the sources. Many approaches use a Hidden Markov Model (HMM), which is strongly related to Chromosome Painting to assign genomic segments to specified reference populations by exploiting LD

Current implementations may scale to the thousands (see Usage) but are limited in scale for learning population structure and are likely to only form a part of a biobank population model when describing external populations. Additionally, the biological parameters needed (e.g., genetic maps, recombination and mutation rate, average ancestry coefficients and average number of generations since admixture) may be unknown and are difficult to learn [131]. A considerable effort for biobanks would be required to store, report and use the per-SNP ancestry information returned.

### Describing genetic variation with an external reference

#### Markers for ancestry and projecting PCs

The use of AIMs to represent genetic diversity within a biobank is not well developed. Because AIMs themselves are indicative, but not diagnostic, of a particular population and are a biased sample of the genome (towards ancestry), it is hard to arrive at an ancestry mixture or other measure of structure. However, with efforts in calibration for external datasets, the information required to assess large-scale structures is clearly present in AIMs, which are standard in all commercial microarrays [132].

It is straightforward to project an individual into the genetic variation of a reference dataset when the reference is described by Principal Components. Associated with each SNP and PC is a weighting, and these must simply be summed. This approach is common in the study of ancient populations, which, due to the high missingness of their data, are often described in terms of modern variation [133].

This has not been performed for biobanks because they contain large variations. However, as discussed above, a meta-analysis of many small populations leads to incomplete correction for stratification. Since there is no standard reference, the results of the projection would also be dependent on the choice of the reference populations. Thereby, they can be easily manipulated and are not comparable across studies [75].

#### Mixtures of known populations

The ancestry models described above can all be structured to allow comparison of a sample dataset with respect to a reference dataset. *ADMIXTURE* [134] is the most popular tool to make “supervised” inferences in this way.

When an individual receives ancestry from different sources, they inherit SNPs and haplotypes in proportion to their ancestry from each source. Therefore, significant power can be obtained by considering not only SNPs but also haplotypes, quantified either by IBD, Chromosome Painting, or some other technique. These methods describe kinship or haplotype sharing with the reference. This, in turn, can be used to learn an individual’s ancestry mixture, which is routinely done, for example, via Non-Negative Least Squares (NNLS) [135, 136] or *SOURCEFIND* [137]. Because the computational cost of these approaches is linear in the size of the target dataset, they can be used at the biobank scale. However, the value of the resulting mixture has yet to be established.

#### Gene pool models

Frequently, we do not have samples from the underlying ancestral components that led to modern populations. “Gene pool” models allow inferred putative ancestral populations to be used in place of fixed reference populations. Ancestral populations are first generated from the allele frequencies of a worldwide panel of individuals that correspond to chosen *K* splits, produced by *ADMIXTURE* or alike program. These “populations” correspond to the putative ancestral populations of all individuals in the dataset. The advantage of creating these populations from a diverse panel of global individuals is that they can be used as a reference to infer the admixture components (e.g., through a *supervised ADMIXTURE*) of other individuals without changing the model. The admixture components can be used to correct for population stratification [58] in the same manner as principal components are used, accepting that they model admixture directly, whereas PCA does not. This approach, first employed for biogeography [36], has been routinely used in population genetic investigations and was shown to be applicable to both modern and ancient populations [34, 138, 139]. Despite its promise, it is yet to be implemented in biobanks; the barriers resemble those of Mixture Models in that a “correct” set of gene pools is hard to establish.

#### Local ancestry models

A local ancestry model can be defined by constructing a reference dataset and applying the local ancestry models to identify ancestry structures within the reference. These approaches have not been widely applied to biobanks in the past due to issues of scale. However, as with the genome-wide haplotype approaches, local ancestry can be learned at scale – efficient approaches scale linearly in the biobank size.

Local genomic ancestry tools are typically used to investigate ancestry on a granular scale, which is necessary when analysing highly admixed individuals, such as African Americans, Latinos or Ashkenazic Jews [33, 42]. The genomes of these individuals constitute a mosaic of geographically and genetically distinct ancestral populations, and local ancestry tools aim to identify the chromosomal boundaries associated with each ancestral population.

However, the promise of comparing to a standard reference simultaneously allows the methods to scale sufficiently and allows comparison across datasets. The key unsolved questions, above those for unlinked methods, are around value. This approach generates extremely large datasets of ancestry information potentially at each SNP. Storing and exploiting such information is a considerable ongoing challenge. Would a fine-scale representation of ancestry help understand the distribution of traits? Does it replace, or complement, the simpler approach of representing ancestry as a proportion of the genome?

### Usage

#### Markers for ancestry

AIMs are identified by finding SNPs that are associated with particular populations or geographic regions. Although many sets of AIMs have been published [109], they were obtained from a handful of populations and their specificity was not validated on other populations. To identify AIMs, it is critical to first assemble a worldwide panel of populations. The search for AIMs is typically performed genome-wide. The putative AIMs should then be evaluated for their specificity and sensitivity in identifying a fine population structure, ideally using a different panel [140]. Finally, global ancestry tools can average the ancestry of each contributing population across the individual’s AIMs and report the average proportion contributed by each ancestral or parental population.

#### Ancestry as a mixture

Global genomic ancestry tools can be categorised as shown in (Figure S1) (Table S1). Whilst *STRUCTURE* was initially the most popular approach, it suffered from several disadvantages. First, its accuracy and reliability have been a source of concern [141, 142]. When the diversity of the native population is low, *STRUCTURE* was shown to produce particularly misleading results [142]. Finally, *STRUCTURE* is a notoriously slow tool, which was soon replaced by dramatically faster implementations.

*FRAPPE* and *ADMIXTURE* are based on a similar approach to *STRUCTURE*, but both use a maximum likelihood estimation approach to optimise the likelihood for allele frequencies and group memberships, using slightly different algorithms. By default, *ADMIXTURE* uses a block relaxation algorithm that allows for fast convergence and highly accurate parameter estimates [143] and has an optional Expectation-Maximisation (EM) algorithm. *FRAPPE* uses solely the EM algorithm [144], which optimises the likelihood for both allele frequencies and fractional group memberships [144]. *FRAPPE* has been demonstrated to not only be much more computationally efficient than *STRUCTURE* but also to produce significantly fewer biased estimates [144]. However, due to its strict convergence criteria, its EM algorithm is computationally intensive and slower than *ADMIXTURE* [143], which was reported to have higher accuracy than *STRUCTURE* and *FRAPPE*.

Spatial approaches, exemplified by *GENELAND* [145, 146], *TESS* [147] and *BAPS* [148], are conceptually similar to *STRUCTURE* but consider geographical coordinates in their prior distributions, allowing identification of the spatial location of genetic variants between populations. Therefore, these software not only group individuals genetically into clusters but are also able to estimate the spatial distribution of these clusters [145–148]. Mitigating privacy concerns has the advantage of replacing a real location with a genetically induced one. Yet the approaches are currently rather inaccurate (perhaps due to population structure being more complex than a simple mixture). There are also no scalable implementations.

Bayesian clustering models have been known to have different strengths and weaknesses that depend on the spatial genetic patterns present and on factors such as gene flow, dispersal distance and demography. *GENELAND* [145, 146] has been demonstrated to be highly efficient when gene flow is low and genetic discontinuities correspond to simple shaped boundaries [149–151]; however, it is sensitive to the level of genetic differentiation [152, 153], and its accuracy [150, 154] and speed in analysing large datasets [145, 146] were criticised. Alternative tools like *TESS* and *BAPS* were shown to outperform *GENELAND* and each other under some scenarios but not in others [145, 146, 148, 154]. Interestingly, Bayesian clustering models are known to overestimate genetic structure in the presence of IBD [151, 155], which highlights the importance of accounting for other types of structure in the data such as cryptic relatedness. Attention should be given to the priors used in Bayesian analyses and their effect on the final results [156].

#### Local ancestry and haplotypes

Local ancestry and haplotype tools can be divided into four categories. Here, we will discuss the four most popular tools (Figure S2): *HAPMIX, ChromoPainter, LAMP and LAMP-LD* (Table S2).

*HAPMIX* [157] is a popular approach that was limited to only two source populations and is unsuitable to biobanks. The biological parameters that *HAPMIX* requests (e.g., genetic maps, recombination and mutation rate, average ancestry coefficients and the average number of generations since admixture) are typically unknown [131]. *MOSAIC* [158] places an HMM over the haplotype estimation performed by *ChromoPainter* to learn how frequently haplotypes from different ancestries appear in unadmixed ancestries. It is, therefore, plausible to run at biobank scales in principle, though considerable effort would be required to report and use the per-SNP ancestry information returned.

*LAMP* and *LAMP-LD* work effectively with three-way admixture and gain a computational advantage by ascribing ancestry to pre-defined windows, though neither scales beyond hundreds of samples or tens of thousands of SNPs [159] and are hence both inapplicable for biobanks [131].

*ChromoPainter* is part of the *fineSTRUCTURE* pipeline [116], which allows the identification of fine-scale population structure that cannot be identified by PCA or related approaches [129]. Chromosome Painting is applicable for samples up to thousands but cannot be used at a biobank scale [130] to examine variation within a sample. It can, however, be used to compare large biobank datasets to standardize references. There is an unpublished fast approximation in the *PBWT* package [160] that can handle hundreds of thousands of samples for analysing within-sample variation.

These methods allow characterisation of LAI and gain power and resolution through analysis of haplotypes. One typical assumption is that admixture tract lengths are independent and exponentially differentiated; therefore, they are less effective when the admixture is strong because the admixture tracts are longer than expected under an exponential distribution [161]. Further, many require phased data and are therefore susceptible to phasing errors.

Overall, the popular local ancestry tools are positioned along the extreme ends of limited models. At one end are mostly HMM-based tools, that either do not consider LD or are limited to two or three reference populations. At the other end are more robust tools that aim to identify haplotypes, but their high memory consumption limits their usage. An additional limitation of the local ancestry approach is the challenging evaluation of the results in follow-up analyses. The local ancestry approach should be preferred when the loci or region of interest are known; however, in an exploratory GWA or MR analyses, it is unclear how to analyse a large number of segments associated with various ancestral populations. In this case, grouping the ancestral populations into geographical regions may be an appropriate compromise between accuracy and power considerations.

#### Sibship, kinship and clanship

Relatedness inference tools exploit different statistical approaches in analysing IBD segments and identifying the correct level of relatedness. We will discuss the six most popular tools: *PLINK, KING, fastIBD, GERMLINE, PC-Relate* and *REAP* (Figure S3) (Table S3).

Kinship can be inferred by kinship coefficient estimation or IBD detection. Kinship coefficient is a classic measurement of relatedness and can be defined as the probability that two homologous alleles are drawn from each of two individuals are identical by descent [127, 128]. Software that estimate the kinship coefficient often use relatedness estimators to calculate the kinship coefficient, which falls into two categories based on the method that they use: likelihood approach to determine the likelihood of a pair of individuals having a relationships (e.g., half-sibs, full-sibs, etc.) and a relatedness-based approach to evaluate the probability of IBD [162].

ML estimators mostly use an EM algorithm to estimate the *K* coefficients (the proportion of genome at which two individuals share 0, 1, or 2 IBD genes); *KING*, *REAP* and *PC-Relate*, use the statistical method of moments to estimate the realised *k* coefficients [163]. *KING* can produce reliable inferences for large sample sizes (millions of unrelated and thousands of relative pairs) [164]. However, *KING* is prone to biased estimates in admixed populations and in the presence of population structure due to the violation of simplifying assumptions that do not hold in the presence of population structure and/or ancestry admixture [165]. Conversely, *REAP* [166] and *PC-Relate* [165] are able to account for different ancestry backgrounds of admixed individuals by using individual-specific allele frequencies derived from model-based population structure analysis methods (e.g., *ADMIXTURE)*. Bias in these allele frequencies can lead to significantly biased relatedness estimates [165]. Despite this, *PC-Relate* has an advantage over *KING* and *REAP*, because they, unlike *PC-Relate*, have difficulty separating unrelated individuals from more distantly related ones [126]. Both tools have relatively high accuracy for first through third-degree classification; however, their accuracy decreased substantially to below 50% for fourth through seventh and unrelated classification [167]. Overall, PC-Relate appears to be the most robust kinship coefficient estimation tool when compared with *KING* and *REAP* due to its ability to work effectively with admixed populations whilst also being able to distinguish between unrelated individuals and more distantly related ones.

Methods for IBD detection identify the similarity between haplotypes that are statistically unlikely to occur in the absence of IBD sharing [168]. *PLINK* [169] incorporates a method of moments approach, using an HMM to infer underlying IBD in chromosomal segments based on observed IBS states. *PLINK* was criticised for producing a high level of false positives (individuals who are unrelated based on IBS sharing but are called as related) for second-degree relationships [170]. *fastIBD* [171] and *GERMLINE* [172] detect “seeds” of identical short haplotype matches and extend them to nearby sites. *fastIBD* can be applied to large sample sizes across genome-wide SNP data; however, it is obliged to carry out haplotype phasing and is therefore susceptible to phasing errors, particularly if the SNP set is small. Computer memory capacities may also limit the number of individuals that can be phased at one time; therefore, in practicality, it is computationally unfeasible to analyse over 100,000 individuals. Whereas *fastIBD* is based on shared haplotype frequency, *GERMLINE* is based on shared haplotype length.

Ramstetter et al. [167] tested the accuracy of relationship inference software on SNP data of large Mexican American pedigrees spanning up to six generations. They showed that there are no “one size fits all” IBD tools and that tools vary in their sensitivity to the IBD segment length, which corresponds to the degree of relatedness. The main reason for this is that haplotype-based IBD segment detection methods struggle to detect long IBD segments if the shared haplotype has discordant alleles due to genotype or phasing error. One solution is to use tools like *Refined IBD* [173] and recover the long IBD segment by mending smaller ones using an external tool [173]. Concurrent methods generally rely on diploid genotype data, which makes them ineffective when dealing with ancient data which have a low concentration of endogenous DNA and fragmentation [125]. Since all tools underperformed in inferring remote relatedness (over 3rd degree) in diverse samples [167], further efforts should be made towards the development and testing of more robust tools.

## Conclusions

The rise of genomic biobanks and biological and computational biotechnology advancements have allowed for significant developments in the field of personalised medicine, making the vision of targeted therapies, accelerated diagnosis and early disease detection become more of a reality. However, the geographic differentiation of human genetic variation (population genetic structure) suggests that the frequencies of certain disease-causing genetic variants, and variants in drug-metabolising genes may differ depending on geographic location, leading to geographic disparities in the susceptibility of an individual to a disease and/or specific drug treatment. Therefore, the lack of representation of diversity in genomic studies poses a limitation in the current global understanding of disease risk and intervention efficacy.

It is widely accepted that increased samples from a much more diverse range of populations is required. However, diversity needs to be quantified, compared and annotated within and between biobanks in order to lead to insight. Biobanks must therefore contain genetic annotations that are comparable, computable and compatible across datasets. Whereas previous studies explored the applicability of bioinformatics tools for association studies (e.g., [55]), this review focussed on whether tools are conceptually comparable and whether they scale. Therefore, it assesses the confounding effects of stratification bias through the identification of population genetic structure in a standardised and comparable manner with a goal of improving biobanks, increasing the accuracy of association analyses and informing developmental efforts. These tools vary in their strengths and limitations; therefore, it is vital to review these characteristics in order to apply them appropriately.

Genomic ancestry inference encompasses tools that are able to identify the ancestry of an individual by utilising specialised markers to compare the genetic similarity of an individual’s DNA to other individuals sampled from a variety of populations or geographic regions. Global genetic ancestry tools assess the average proportion contributed by each ancestral population across the whole of the individual’s genome, whilst local ancestry inference tools identify the ancestry of distinct segments within chromosomes.

Simple descriptions such as Ancestry Informative Markers (AIMs) and Low Dimensional Representation with PCA are useful but insufficient. Current best-practice includes correcting for kinship using the Genetic Relatedness Matrix (GRM) which may be valuable but does not provide a framework for interpreting external datasets.

For global genetic ancestry inference, some tools do scale well enough to be considered for biobanks. The limitations include unrealistic assumptions, a tendency to mistake cryptic relatedness for genetic structure, conceptual issues in the interpretation of admixture, and a lack of prior research into how global ancestry can be usefully applied for association studies.

GRM approaches can jointly represent population genetic structure and cryptic relatedness, which can avoid consequent false-positive associations in GWAS within a single dataset. More fine-scaled representations exist in the form of kinship (measuring IBD) and haplotype similarity (Chromosome Painting) matrices, which are scalable. In all cases, these capture an inherently noisy and hence statistical property. Consequently, further efforts should be made towards the development of more robust tools for remote degrees of relatedness (over 3rd degree) in diverse samples, especially in the case of cross-dataset comparison. Studies are needed in order to explore the value of these fine-scale approaches for biobanks.

Local ancestry inference tools are still slower, though they can be deployed similarly to phasing and imputation should a compelling use case be found. Efforts should be made to develop a new approach that addresses the common limitations, including a requirement of phased data and consequent susceptibility to phasing errors, ability to model LD, and restrictions in terms of the number of populations. The local ancestry approach is clearly deployable when the region of interest is known. It may be useful to group the ancestral populations by geographic region as a way of compromising between accuracy and power considerations. Correctly deployed, local ancestry could correct for local genetic correlations in a way that is much more powerful than simple correlations as captured by LD, though the value for association studies is yet to be determined.

Furthermore, the rise of paleogenomic medicine and rapid accumulation of ancient genomes have already shed light on several conditions (e.g., [174] also requires the development of specialised kinship inference software that are capable of handling ancient DNA). At the moment, however, most current methods rely on diploid genotype data making them ineffective when handling ancient DNA.

With rapid advances in technology and the dense amount of genetic variation data available, we can continue to expect the development of new inference software and enhancements of existing ones. For example, there is much scope for improved modelling of LD to reduce error rates and improve the ability to detect subtle population structures. However, a challenge for the future will be to develop inference methods that are computationally efficient and applicable to large sample sizes whilst being able to fully exploit the rich information available in the form of haplotypes. There is also currently a lack of representation of non-European populations in genetic studies. Unless populations of diverse ancestries are included, therefore incorporating an equal knowledge of genetic variation across ancestry groups, it could contribute further to health disparities and negatively impact genomic interpretation. Efforts should be made to include data from more diverse populations in GWAS and develop robust population structure models that can reduce or eliminate the *stratification bias* from the cohort. Not only this, but biobanks must begin to incorporate individual-level genetic annotations that are comparable, computable and compatible across datasets. Clinicians must also be properly trained to understand their output so that they can make an informed decision as to whether or not a genetic variant may be causative or whether the association is likely the result of population stratification.

Overall, with increased availability of large genomic datasets, an equal representation of genetic variation across ancestry groups and continuous improvement and development of genetic inference software, population structure inference will occur with finite detail. This will allow for more effective differentiation between closely related populations, in turn allowing for individual-level genetic annotations to be incorporated in biobanks and increasing the accuracy of association analyses.

In summary, we have identified a gap in the literature concerning the design and standardization of biobanks. Started as localised initiatives, the progress in sequencing technologies sparred the rapid growth of biobanks in size, diversity and geography, although conceptually they are still thought of as local datasets. This perception limits the usefulness of biobanks and prevents banking on their resources in joint analyses. To overcome this limitation, it is critical to develop a holistic solution to the problem of population structure. Current strategies implemented in the various tools aim to expose different aspects of the data by ubiquitously mapping the ancestry of individuals, though none could be used as a complete solution to ancestry. One unsolved challenge is to create representations that are useful for meta-analysis without sharing individual-level data. Natural summaries of admixtures can be created from means and variances, but it is an open question to establish whether these are sufficiently accurate and whether alternative representations can protect privacy whilst maximising research benefits. PCA’s accuracy, in specific, has been challenged by several groups. Yet other tools also suffer from limitations related either to their design, which affect their speed and accuracy, or their basic assumptions concerning human populations, which, in turn, affect the usefulness of their output to the population genetics. These shortcomings, often unacknowledged, limit our ability to interpret the results and increase the burden of evidence when using these tools. Further efforts should be made to explore the limitations of these tools and optimal usage on global and massive datasets as well as to divide new approaches that overcome the most common limitations of running time, identification of admixture and high specificity among human populations.

We end this review with five take-away messages: firstly, more diverse data are needed worldwide, both from populous populations who will benefit from inclusion in datasets *en masse,* as well as pockets of genetic diversity that may shed light on biological processes that would otherwise remain undiscovered. Secondly, the methodology to interpret and harmonise results from diverse datasets is not ready. Thirdly, the main barrier is in the creation of shareable and comparable summary statistics from diverse data. Fourthly, these summary datasets should be carefully designed to allow effective association correction, as well as meta-analysis, which we argue requires placing the genetics into some type of model. Finally, clinicians, geneticists and epidemiological researchers will all have to learn how to exploit the information that comes from the genomic diversity revolution when it comes.

As this review is written at the height of the COVID-19 pandemic and biobank data are internationally shared to improve diagnosis and treatment outcome, practically transforming our vision of international biobanks into reality, we hope that our study would serve to improve the accuracy, reliability and replicability of association studies and biobanks.

## Supplementary Information

**Additional file 1: Text S1**. concepts used in the study. **Table S1**. A summary of the advantages and limitations of seven of the most popular global genomic ancestry tools, as discussed in this review. **Table S2**. A summary of the advantages and limitations of the five of the most popular selected local ancestry inference tools. **Table S3**. A summary of the advantages and limitations of the six most popular of the selected relatedness inference tools, as discussed in this review. **Figure S1**. The popularity (normalised number of citations) of the different GAI software, separated into model-based (red) and non-parametric (blue) tools. The tools were found using existing review papers and free search, using the search engines ‘Google,’ ‘Google Scholar’ and the journal ‘Bioinformatics’ to search for keywords including: ‘software,’ ‘tools,’ ‘inference,’ ‘biogeographic,’ ‘ancestry,’ ‘kinship’ and ‘haplotype.’ The number of citations for the paper proposing the tool, taken from ‘Google Scholar,’ were compared for each tool within each domain. To account for the differences in the number of years since publication, the number of citations was normalised by dividing the number of citations by the number of years since publication. **Figure S2**. The popularity (normalised number of citations) of the different LAI software, separated into their technologies: Hidden Markov Model (HMM) (green), Chromosome Painting (red) and Statistical Learning Algorithm (SLA) (blue). Finding the tools and calculating the normalised citation number was done as in Figure S1. **Figure S3**. The popularity (normalised number of citations) of the different kinship inference software, separated into their software strategies: Identity-By-Descent (IBD) detection (red) and Kinship Coefficient Estimation (blue). Finding the tools and calculating the normalised citation number was done as in Figure S1

## Data Availability

All our data and materials are publicly available.

## Abbreviations

AIM: Ancestry Informative Marker
EM algorithm: Expectation-Maximisation algorithm
GAI: Global Ancestry Inference
HMM: Hidden Markov Model
MCMC: Markov Chain Monte Carlo
MLE: Maximum Likelihood Estimate
MLM: Mixed Linear Model
LMM: Linear Mixed Model
MDS: Multidimensional Scaling
PCA: Principal Components Analysis
GWAS: Genome-Wide Association Study
BMI: Body-Mass Index
SNP: Single Nucleotide Polymorphism
RCT: Randomised Controlled Trial
MR: Mendelian Randomisation
LD: Linkage Disequilibrium
IBD: Identical By Descent
IBS: Identical by State
LAI: Local Ancestry Inference
