# Supplementary material for: Population genetic considerations for using biobanks as international resources in the pandemic era and beyond
Source: BMC Genomics. 2021 May 17;22:351. doi: 10.1186/s12864-021-07618-x (PMC8127217; doi:10.1186/s12864-021-07618-x)
Supplement: Supplementary file 1 — Additional file 1: Text S1. concepts used in the study. Table S1. A summary of the advantages and limitations of seven of the most popular global genomic ancestry tools, as discussed in this review. Table S2. A summary of the advantages and limitations of the five of the most popular selected local ancestry inference tools. Table S3. A summary of the advantages and limitations of the six most popular of the selected relatedness inference tools, as discussed in this review. Figure S1. The popularity (normalised number of citations) of the different GAI software, separated into model-based (red) and non-parametric (blue) tools. The tools were found using existing review papers and free search, using the search engines ‘Google,’ ‘Google Scholar’ and the journal ‘Bioinformatics’ to search for keywords including: ‘software,’ ‘tools,’ ‘inference,’ ‘biogeographic,’ ‘ancestry,’ ‘kinship’ and ‘haplotype.’ The number of citations for the paper proposing the tool, taken from ‘Google Scholar,’ were compared for each tool within each domain. To account for the differences in the number of years since publication, the number of citations was normalised by dividing the number of citations by the number of years since publication. Figure S2. The popularity (normalised number of citations) of the different LAI software, separated into their technologies: Hidden Markov Model (HMM) (green), Chromosome Painting (red) and Statistical Learning Algorithm (SLA) (blue). Finding the tools and calculating the normalised citation number was done as in Figure S1. Figure S3. The popularity (normalised number of citations) of the different kinship inference software, separated into their software strategies: Identity-By-Descent (IBD) detection (red) and Kinship Coefficient Estimation (blue). Finding the tools and calculating the normalised citation number was done as in Figure S1 [file 12864_2021_7618_MOESM1_ESM.pdf]

SUPPLEMENTARY MATERIALS

Text S1 – concepts used in the study

Admixed Population
A population of individuals with ancestors from two or more relatively distinct populations relatively recent in human history.

Admixture mapping
Mapping of susceptibility alleles for a phenotype that shows differential risk by ancestry with the aim of correlating the ancestry around the genomic region associated with the phenotype.

Bayesian clustering
Assignment of individuals to clusters based on genetic similarity without assuming predefined populations, using statistical methods that allow inferences to be drawn from the data and prior information.

Expectation-Maximisation (EM) algorithm
An iterative method to find maximum likelihood estimates (MLE) of parameters in statistical models, altering between an expectation step (creates a function for the expectation of the log-likelihood evaluated using the current estimate for the parameters) and a maximisation step (computes parameters maximizing the output of the expectation step).

Genetic Relatedness Matrix (GRM)
The GRM represents the genomic similarities among all individuals. Each cell in the matrix measures the genotypic correlation between a pair of individuals (the rows and columns). The GRM can be used with a phenotypic distance matrix to estimate heritability without estimating the phenotypic effect of individual SNPs.

Hidden Markov Model (HMM)
A statistical Markov model is a randomly changing system assumed to consist of future states that only depend on current states, whereby states are unobservable (hidden).

Markov Chain Monte Carlo (MCMC)
A simulation method used in Bayesian calculations, incorporating a class of algorithms that can obtain a sample of the desired distribution by observing several steps of the Markov chain, which is a sequence of a probability of events that depend only on the state of the previous event and has the desired distribution at its equilibrium.

Maximum Likelihood Estimate (MLE)
A method for estimating the parameters of a probability distribution by maximising the likelihood function, where the observed data, under the assumed statistical model, are the most probable.

Maximum optimisation
Maximising a function by choosing input values from a specified set and using this to calculate the value of the function.

Mixed Linear Models (MLM) or Linear Mixed Models (LMMs)
Mixed linear models that incorporate both fixed and random effects where there is non-independence in the data. These models are used as a form of “global correction” as they account for both ancestry

and relatedness.

Multidimensional Scaling (MDS)
A type of multivariate analysis which allows multidimensional information to be displayed graphically (usually two dimensions) with minimum loss of information.

Principal Components Analysis (PCA)
A type of multivariate analysis that reduced the dimensionality of the dataset with minimum loss of information while preserving covariance of data. PCA is typically applied to genotype data as a form of "global correction" and the derived PCs are used for further analyses. PCA represents the reduced dataset as principal components (PCs) that can be displayed on scatter plots.

Table S1: *A summary of the advantages and limitations of seven of the most popular global genomic ancestry tools, as discussed in this review.*

TOOL	DESCRIPTION	SUMMARY
**STRUCTURE** (Pritchard, Stephens and Donnelly, 2000)<br><br>**Citations**: 24,418 (1285.16)	*STRUCTURE* uses a Bayesian clustering approach and applies Markov Chain Monte Carlo (MCMC) estimation, which randomly assigns sampled individuals to *k* groups based on estimations of genetic variant frequencies (Pritchard, Stephens and Donnelly, 2000). In theory, samples belonging to the same cluster can be considered either belonging to the same population or at least sharing a close evolutionary history. *STRUCTURE* was extended later to accommodate LD, permitting the inclusion of weakly linked markers with some degree of non-independence (Falush, Stephens and Pritchard, 2003).	**ADVANTAGES**<br>● User friendly (Pritchard, Stephens and Donnelly, 2000).<br>● Applicable to most common genetic markers (Pritchard, Stephens and Donnelly, 2000).<br><br>**LIMITATIONS**<br>● Very computationally intensive and slower than FRAPPE, ipPCA, BAPS and LAMP (Tang *et al.*, 2005; Sankararaman *et al.*, 2008; Intarapanich *et al.*, 2009).<br>● Difficulty resolving ancestry clusters containing a small number of individuals (Intarapanich *et al.*, 2009).<br>● Interval and standard error estimation are costly (Pritchard, Stephens and Donnelly, 2000).<br>● Less accurate than LAMP, ipPCA and BAPS (Sankararaman *et al.*, 2008; Intarapanich *et al.*, 2009).<br>● High risk of misleading results when used in the context of introduction route inference (Lombaert, Guillemaud and Deleury, 2018).<br><br>**ASSUMPTIONS**<br>● Models LD (Pritchard, Stephens and Donnelly, 2000).<br>● Hardy–Weinberg equilibrium (HWE) (Pritchard, Stephens and Donnelly, 2000).<br>● Fixed number of populations (Pritchard, Stephens and Donnelly, 2000).<br>● Marker loci are unlinked and at linkage equilibrium (Pritchard, Stephens and Donnelly, 2000).<br><br>**RUNNING TIME**<br>Analysing 307 individuals from 14 geographic locations using a 2.3-GHz processor took more than 3 days in *STRUCTURE* (Intarapanich *et al.*, 2009).<br><br>Analysing 500 individuals from 2 ancestral populations using a 2-GHz processor took 5 hours in *STRUCTURE* versus 1 minute in *FRAPPE* (Tang *et al.*, 2005).
**ADMIXTURE** (Alexander, Novembre	*ADMIXTURE* is based on a similar approach to *STRUCTURE*. Still, like *FRAPPE*, it uses a maximum likelihood estimation approach to	**ADVANTAGES**<br>● Block relaxation algorithm that allows for fast convergence and highly accurate

and Lange, 2009)<br><br>**Citations:** 2,111 (211.10)	optimise the likelihood for allele frequencies and group memberships, using slightly different algorithms. By default, *ADMIXTURE* uses a block relaxation algorithm that allows for fast convergence and highly accurate parameter estimates (Alexander, Novembre and Lange, 2009) and has an optional Expectation-Maximisation (EM) algorithm.	parameter estimations (Alexander, Novembre and Lange, 2009).<br>● Faster and more accurate than *STRUCTURE* and *FRAPPE*, which allows analyzing thousands of individuals across thousands of genetic markers (Alexander, Novembre and Lange, 2009).<br><br>**LIMITATIONS**<br>● Bootstrap resampling to compute standard errors takes a long time (Alexander, Novembre and Lange, 2009).<br>● Difficulty resolving ancestry clusters containing a small number of individuals (Alexander, Novembre and Lange, 2009).<br>● Parameters of the *ADMIXTURE* model must satisfy linear constraints and bounds (Alexander, Novembre and Lange, 2009).<br>● Limited when using samples of ancient DNA due to violation of HWE assumption (Joseph and Pe'er, 2018).<br><br>**ASSUMPTIONS**<br>● HWE (Alexander, Novembre and Lange, 2009).<br>● Linkage equilibrium among markers (Alexander, Novembre and Lange, 2009).<br><br>**RUNNING TIME**<br>For 10 simulated datasets with 10,000 markers, the supervised analysis took an average of 5.15 seconds, while unsupervised analysis averaged 27.5 seconds (Alexander and Lange, 2011).
**FRAPPE** (Tang *et al.*, 2005)<br><br>**Citations:** 476 (34.00)	*FRAPPE* is based on a similar approach to *STRUCTURE*, but like ADMIXTURE, it uses a maximum likelihood estimation approach to optimise the likelihood for allele frequencies and group memberships, using slightly different algorithms. *FRAPPE* uses solely the EM algorithm, which is used to optimise the likelihood for both allele frequencies and fractional group memberships (Tang *et al.*, 2005).	**ADVANTAGES**<br>● More robust than *ADMIXTURE* (faster and more efficient (Yuan *et al.*, 2017)<br>● Faster than *STRUCTURE* (Tang *et al.*, 2005).<br>● Does not require phased ancestral data and is therefore not prone to phasing errors (Tang *et al.*, 2005).<br><br>**LIMITATIONS**<br>● The strict convergence criteria of its EM algorithm makes it computationally intensive and slower than *ADMIXTURE* (Alexander, Novembre and Lange, 2009).<br>● Loosening the convergence criteria of the EM algorithm allows faster termination of the EM algorithm but jeopardises the accuracy of estimates (Alexander, Novembre and Lange, 2009).<br><br>**ASSUMPTIONS**<br>• Linkage equilibrium among markers (Tang *et al.*, 2005).

<table>
<tr><td></td><td></td><td>RUNNING TIME
Analysing 500 individuals from 2 ancestral populations using a 2-GHz processor took 1 minute in FRAPPE (Tang et al., 2005).</td></tr>
<tr><td>GENELAND
(Guillot, Mortier and Estoup, 2005)

Citations:
984 (70.29)</td><td>Spatial approaches, exemplified by GENELAND (Guillot, Mortier and Estoup, 2005), TESS (Durand et al., 2009) and BAPS (Corander, Waldmann and Sillanpää, 2003) are conceptually similar to STRUCTURE, but consider geographical coordinates in their prior distributions, allowing identification of the spatial location of genetic variants between populations. These software do not only group individuals genetically into clusters but can also estimate the spatial distribution of these clusters (Corander, Waldmann and Sillanpää, 2003; Guillot, Mortier and Estoup, 2005; Durand et al., 2009).

GENELAND makes use of georeferenced individual multilocus genotypes for the inference of the number of populations and of the spatial location of genetic discontinuities between those populations.
GENELAND can be used to detect population structure in the form of systematic variation of allele frequency, which can be detected from departure from Hardy-Weinberg and linkage equilibrium. GENELAND requires individual multilocus genetic data that are optionally georeferenced, and implements several models that can make use of both geographic and genetic information to estimate the number of populations in a dataset and delineate their spatial organisation (Guillot, Mortier and Estoup, 2005).</td><td>ADVANTAGES
● Can identify the spatial location of genetic variants between populations (Guillot, Mortier and Estoup, 2005).
● User friendly (Guillot, Mortier and Estoup, 2005).
● Flexible to extend (Guillot, Mortier and Estoup, 2005).

LIMITATIONS
● Outperformed by TESS and BAPS in terms of accuracy and misassignment rates (Latch et al., 2006; Chen et al., 2007).
● Does not handle admixture (Guillot, Mortier and Estoup, 2005).
● Computationally intensive (Guillot, Mortier and Estoup, 2005).

ASSUMPTIONS
● HWE (Guillot, Mortier and Estoup, 2005).
● Linkage equilibrium among markers (Guillot, Mortier and Estoup, 2005).
● Known allele frequencies (Guillot, Mortier and Estoup, 2005).

RUNNING TIME
GENELAND is computationally intensive with large datasets (Guillot, Mortier and Estoup, 2005), taking roughly 20 minutes to analyse 500 individuals with 20 loci using a 2-GHz processor (Corander et al., 2003).</td></tr>
<tr><td>BAPS
(Corander, Waldmann and Sillanpää, 2003)

Citations:
582 (44.77)</td><td>Spatial approaches, exemplified by GENELAND (Guillot, Mortier and Estoup, 2005), TESS (Durand et al., 2009) and BAPS (Corander, Waldmann and Sillanpää, 2003) are conceptually similar to STRUCTURE, but consider geographical coordinates in their prior distributions, allowing identification of the spatial location of genetic variants between populations. These software do not only group individuals genetically into clusters but are also able to estimate the spatial distribution of these clusters (Corander, Waldmann and Sillanpää, 2003; Guillot, Mortier and Estoup, 2005; Durand et al., 2009).</td><td>ADVANTAGES
● Can identify the spatial location of genetic variants between populations (Corander, Waldmann and Sillanpää, 2003).
● Outperforms GENELAND in terms of accuracy and misassignment rates (Latch et al., 2006; Chen et al., 2007).

LIMITATIONS
● Memory intensive (Corander, Waldmann and Sillanpää, 2003).
● Slower and less accurate than ipPCA (Intarapanich et al., 2009).
● Lower accuracy than GENELAND at low levels of genetic differentiation (Latch et al.,</td></tr>
</table>

	BAPS combines a discrete parameterization of admixture proportions in genomes with a simulation framework that yields a clear biological interpretation of the estimation results and can be used to assess the statistical significance of putative admixture events. The number of genetically differentiated sources contributing to a dataset is inferred first using a mixture model. Thereafter, given such an estimate, admixture events can be learned on a more stable basis using a Monte Carlo simulation-based algorithm (Corander, Waldmann and Sillanpää, 2003).<br><br>*BAPS* treats both allele frequencies of the molecular markers (or nucleotide frequencies for DNA sequence data) and the number of genetically diverged groups in the population as random variables. However, analyses and model comparisons can also be performed using a fixed number of genetically diverged groups or pre-specified population structures (Corander, Waldmann and Sillanpää, 2003).	2006).<br><br>**ASSUMPTIONS**<br>● HWE (Corander, Waldmann and Sillanpää, 2003).<br>● Linkage equilibrium among markers (Corander, Waldmann and Sillanpää, 2003).<br><br>**RUNNING TIME**<br>The authors of *BAPS* note that their software took just 30 minutes to compute an entire sequential admixture estimation for complete human data (Corander, Waldmann and Sillanpää, 2003).
**TESS**<br>(Durand *et al.*, 2009)<br><br>**Citations:**<br>292 (22.46)	Spatial approaches, exemplified by *GENELAND* (Guillot, Mortier and Estoup, 2005), *TESS* (Durand *et al.*, 2009) and *BAPS* (Corander, Waldmann and Sillanpää, 2003) are conceptually similar to STRUCTURE, but consider geographical coordinates in their prior distributions, allowing identification of the spatial location of genetic variants between populations. These software do not only group individuals genetically into clusters but also estimate the spatial distribution of these clusters (Corander, Waldmann and Sillanpää, 2003; Guillot, Mortier and Estoup, 2005; Durand *et al.*, 2009).<br><br>*TESS* implements ancestry estimation algorithms for spatial population genetic analyses. The program performs individual geographical assignment and admixture analysis and can run genome scans for selection. *TESS* is particularly suited to seeking genetic discontinuities in continuous populations and estimating spatially varying individual admixture proportions. *TESS* returns graphical displays of geographical cluster assignments or admixture	**ADVANTAGES**<br>● Can identify the spatial location of genetic variants between populations (Durand *et al.*, 2009).<br>● Outperforms *GENELAND* and *BAPS* in terms of accuracy and misassignment rates (Latch *et al.*, 2006; Chen *et al.*, 2007).<br>● Provides admixture and none admixture models (Durand *et al.*, 2009).<br><br>**LIMITATIONS**<br>• Highest misassignment rates compared with *BAPS* and *GENELAND* (Basto *et al.*, 2016).<br><br>**ASSUMPTIONS**<br>● HWE (Durand *et al.*, 2009).<br>● Parental populations are assumed to be in migration/drift equilibrium (admixture model) (Durand *et al.*, 2009).

	proportions (depending on the model used) and textual output of the admixture Q matrix (Durand *et al.*, 2009).	
**EIGENSTRAT** (Price *et al.*, 2006)<br><br>**Citations:** 6,859 (527.62)	The PCA of *EIGENSTRAT* aims to reduce the dimensionality of the SNP dataset by reducing the genetic markers into principal components (PCs), the first two of which are typically plotted on a scatter plot. The output is used to identify outliers, assign individuals into populations, match cases and controls and more. PCA is one of the most commonly used tools in population genetics due to its high speed, relatively low computational demands, ease of use and appealing graphical results (Patterson *et al.*, 2004).<br><br>*EIGENSTRAT* consists of three steps. First, PCA is applied to genotype data to infer continuous axes of genetic variation, which reduces the data to a small number of dimensions, describing as much variability as possible. In datasets with ancestry differences between samples, axes of variation often have a geographic interpretation. Second, genotypes and phenotypes are continuously adjusted by amounts attributable to ancestry along each axis via computing residuals of linear regressions, creating a virtual set of matched cases and controls. Finally, association statistics are computed using ancestry-adjusted genotypes and phenotypes (Price *et al.*, 2006).	**ADVANTAGES**<br>● Faster than *STRUCTURE* (Price *et al.*, 2006).<br>● Memory efficient and applicable to large genomic scale (Price *et al.*, 2006).<br>● Output can be used to produce intuitive plots (Price *et al.*, 2006).<br><br>**LIMITATIONS**<br>● Difficulty resolving ancestry clusters containing a small number of individuals (Price *et al.*, 2006).<br>● Ineffective when analysing closely related sub-populations (Intarapanich *et al.*, 2009).<br>● Cannot account for admixture (Elhaik and Ryan, 2019)<br>● Uneven sample size can bias results (McVean, 2009).<br>● Most importantly, PCA results may not be reliable, robust, or replicable, as is commonly assumed. PCA should thereby not be used for population genomic analyses. (Elhaik 2021).<br><br>**ASSUMPTIONS**<br>NA.<br><br>**RUNNING TIME**<br>Less than 15 minutes to analyse a dataset with 1,000 samples and 100,000 makers (Price *et al.*, 2006).

Table S2: *A summary of the advantages and limitations of the five of the most popular selected local ancestry inference tools.*

TOOL	DESCRIPTION	SUMMARY
**HAPMIX**<br>(Price *et al.*, 2009).<br><br>**Citations (normalised):**<br>414 (41.40)	*HAPMIX* can be used to accurately infer chromosomal segments of distinct ancestry in admixed populations using dense genetic data. *HAPMIX extends the* model of Li and Stephens, estimating the likelihood of a haplotype (alleles at multiple SNPs) belonging to each reference population. The analysis of haplotypes accounts for LD and allows differentiation of very closely related populations (Price *et al.*, 2009).<br><br>At each position in the genome, *HAPMIX* estimates the likelihood that a haplotype from an admixed individual is a better statistical match to one reference population or the other. A Hidden Markov Model (HMM) is used to combine these likelihoods with information from neighboring loci to provide a probabilistic estimate of ancestry at each locus (Price *et al.*, 2009).	**ADVANTAGES**<br>● Does not require phasing of data (Price *et al.*, 2009).<br>● Computationally tractable, even with large datasets (provided a cluster of computing nodes available) (Price *et al.*, 2009).<br>● Works effectively with unphased data and allows for some miscopying of ancestry segments from the wrong population (Price *et al.*, 2009).<br><br>**LIMITATIONS**<br>● Slower than *LAMP-LD* (Price *et al.*, 2009).<br>● Extremely computationally intensive.<br>● Restricted to two reference populations, limiting accuracy and usefulness (Pugach *et al.,* 2011).<br>● Requires specification of several biological parameters that may be difficult to obtain (Dias-Alves, Mairal and Blum, 2018).<br>● Reduced accuracy when using haplotypes that have been computationally phased (Dias-Alves, Mairal and Blum, 2018).<br><br>**ASSUMPTIONS**<br>● Models LD (Price *et al.*, 2009).<br>● Admixture tract lengths are exponentially distributed and independent (Price *et al.*, 2009).<br>● Limited to two populations (Price *et al.*, 2009).<br><br>**RUNNING TIME**<br>For a dataset of roughly 1,000 admixed individuals on a cluster of 100 nodes, the running time is approximately five hours (Price *et al.*, 2009).
**LAMP**<br>(Sankararaman *et al.*, 2008).<br><br>**Citations (normalised):**<br>271 (24.64)	*LAMP* is a software package for the inference of locus-specific ancestry in recently admixed populations. *LAMP* uses a sliding-windows framework to calculate an optimal window length of contiguous SNPs and then uses a clustering algorithm to estimate ancestry from large reference haplotype data (Sankararaman *et al.*, 2008).<br><br>*LAMP* firstly calculates an optimal window length, on which a clustering algorithm operates and estimates each individual's ancestry. A majority vote is	***ADVANTAGES***<br>● Faster and more accurate than *SABER* and *STRUCTURE* (Sankararaman *et al.*, 2008).<br>● SLA algorithm for optimal window size (Sankararaman *et al.*, 2008).<br>● Robust to phasing errors (Sankararaman *et al.*, 2008).<br><br>**LIMITATIONS**<br>● Does not provide information about LD in ancestral populations (Sankararaman *et al.*, 2008).<br>● Admixture of two populations only (*LAMP-ANC* >2) (Sankararaman *et al.*, 2008).

	then used for each SNP, over all the windows that overlap with the SNP, to decide the most likely ancestral populations at the SNP (Sankararaman *et al.*, 2008).	**ASSUMPTIONS**<br>● Absence of recombination between windows (Sankararaman *et al.*, 2008).<br>● Recombination rates across the genome are known (Sankararaman *et al.*, 2008).<br><br>**RUNNING TIME**<br>*LAMP* analysed 38,864 SNPs in 394 seconds (200 times faster than SABER and 10^4 times faster than STRUCTURE) ((Sankararaman *et al.*, 2008).
**LAMP-LD**<br>(Baran *et al.*, 2012)<br><br>**Citations (normalised):**<br>147 (21.00)	*LAMP-LD* is an extension of the model implemented in the original *LAMP* tool (Sankararaman *et al.*, 2008). *LAMP-LD* integrates a two-layer HMM into the *LAMP* window-based framework to leverage haplotype structure, in turn accounting for LD.<br><br>*LAMP-LD* divides the genome into non-overlapping windows such that no transitions between ancestries are made within each window. Limiting the occurrence of ancestry transitions in this window-based framework improves the inference quality by eliminating extremely short, likely artifactual, ancestral segments (Baran *et al.*, 2012).	**ADVANTAGES**<br>● Robust to phasing errors (Baran *et al.*, 2012).<br>● Faster than HAPMIX (Baran *et al.*, 2012).<br>● Works effectively with three-way admixture (Baran *et al.*, 2012).<br>● Able to more efficiently handle large datasets than LAMP (Baran *et al.*, 2012).<br><br>**LIMITATIONS**<br>● Limited memory allocation and computationally intensive (Dias-Alves, Mairal and Blum, 2018).<br>● Significant reductions in accuracy when analysing sequencing data rather than genotyping data (Brown and Pasaniuc, 2014).<br><br>**ASSUMPTIONS**<br>● Models LD (Baran *et al.*, 2012).<br>● Constant recombination rate (Baran *et al.*, 2012).<br>● No transitions between ancestries are made between windows (Baran *et al.*, 2012).<br><br>**RUNNING TIME**<br>*LAMP-LD* is faster than *HAPMIX,* analysing 200 admixed genotypes in roughly three days compared to *HAPMIX*'s 22 days (1.1-GHz processor) (Baran *et al.*, 2012).
**ChromoPainter**<br>(Lawson *et al.*, 2012)<br><br>**Citations (normalised):**<br>424 (60.57)	Chromosome painting is a related method that does not attempt to identify ancestry tracts but instead identifying the individual who is the most recent common ancestor for every individual at every location in the genome. It "paints" each individuals' genome as a combination of all others to produce a co-ancestry matrix, analogous to the GRM but counting recently shared ancestors instead of SNPs.<br><br>*ChromoPainter* is part of the *fineSTRUCTURE* pipeline (Lawson *et al.*, 2012), which allows the identification of	**ADVANTAGES**<br>● Visualisation of LAI estimates (Lawson *et al.*, 2012).<br>● Can analyse more than two populations (Lawson *et al.*, 2012).<br><br>**LIMITATIONS**<br>● Less effective when the admixture is strong due to admixture tract assumption (Lawson *et al.*, 2012).<br>● Susceptible to phasing errors (Lawson *et al.*, 2012).<br><br>**ASSUMPTIONS**<br>● Models LD (Lawson *et al.*, 2012).

	fine-scale population structure that cannot be identified by PCA or related approaches (Leslie *et al.*, 2015). *ChromoPainter* achieves this by utilising the tree relationship between haplotypes for different genes and considering the time to the most recent common ancestor, which changes along the genome due to recombination resulting in different trees for different genes. It then looks for the haplotypes in the genome that are closest to a haplotype in question, which is then used to paint said haplotype as a combination of all other haplotypes and to produce a co-ancestry matrix. This co-ancestry matrix can then be used for inference in *fineStructure*, which can identify the clusters that differ and assess the statistical differences between them, therefore identifying the potentially different populations (Lawson *et al.*, 2012).	• Admixture tracts are independent and exponentially distributed (Lawson *et al.*, 2012).<br>• Computationally demanding (Lawson *et al.*, 2012).<br><br>**RUNNING TIME**<br>The *ChromoPainter* and *fineSTRUCTURE* pipeline analysed 500 individuals with 3,000-15,000 single-nucleotide variants in roughly 180-500 minutes, whilst taking 25,000-55,000 minutes to analyse 5,000 individuals with 3,000-15,000 SNVs (desktop computer with 32 GB RAM) (Pan *et al.*, 2017).

Table S3: *A summary of the advantages and limitations of the six most popular of the selected relatedness inference tools, as discussed in this review.*

TOOL	DESCRIPTION	SUMMARY
**KING**<br>(Manichaikul *et al.*, 2010).<br><br>**Citations (normalised):**<br>607 (67.44)	*KING* uses moment relatedness estimators to calculate the kinship coefficient, which uses the statistical method of moments to estimate the realised *k* coefficients; the proportion of genome at which two individuals share 0, 1, or 2 IBD genes (Wang, Sverdlov and Thompson, 2016).<br><br>*KING* is a framework for relationship inference combined with a fast algorithm for relationship inference appropriate for use on samples with thousands of individuals genotyped at millions of SNPs from autosomes, consistent with a scale typically achieved in a GWAS. *KING* includes two different methods: (1) *KING*-homo, derived under the assumption of population homogeneity and (2) *KING*-robust, which provides robust relationship inference in the presence of population substructure (Manichaikul *et al.*, 2010).	**ADVANTAGES**<br>● Produces reliable inference even on millions of unrelated pairs of individuals and thousands of relative pairs (Manichaikul *et al.*, 2010).<br>● Performs well under extreme population stratification (Manichaikul *et al.*, 2010).<br>● *KING* can produce reliable inference for large sample sizes (millions of unrelated and thousands of relative pairs) and is also significantly faster than *PLINK* (Manichaikul *et al.*, 2010).<br><br>**LIMITATIONS**<br>● Prone to biased estimates in admixed populations and the presence of population structure (Conomos *et al.*, 2016).<br>● Cannot separate unrelated individuals from distantly related ones (Moltke and Albrechtsen, 2014).<br>● Relatively high accuracy for first through third-degree classification; however, their accuracy decreased substantially to below 50% for fourth through seventh and unrelated classification (Ramstetter *et al.*, 2017).<br><br>**ASSUMPTIONS**<br>• HWE among SNPs with the same underlying allele frequencies (Manichaikul *et al.*, 2010).<br><br>**RUNNING TIME**<br>*KING* was able to analyse a dataset of 180,901 individuals in one minute (3.20-GHz processor) (Manichaikul *et al.*, 2010).
**REAP**<br>(Thornton *et al.*, 2012).<br><br>**Citations (normalised):**<br>135 (19.29)	*REAP* uses moment relatedness estimators to calculate the kinship coefficient, which uses the statistical method of moments to estimate the realised *k* coefficients; the proportion of genome at which two individuals share 0, 1, or 2 IBD genes (Wang, Sverdlov and Thompson, 2016).<br><br>*REAP* appropriately accounts for population structure and ancestry-related assortative mating by using individual-specific allele	**ADVANTAGES**<br>● Can work well with admixed samples (Thornton *et al.*, 2012).<br><br>**LIMITATIONS**<br>● Cannot separate unrelated individuals from distantly related ones (Moltke and Albrechtsen, 2014).<br>● Relatively high accuracy for first through third-degree classification; however, their accuracy decreased substantially to below 50% for fourth through seventh and unrelated classification (Ramstetter *et al.*, 2017).

	frequencies at SNPs that are calculated based on ancestry derived from whole-genome analysis (Thornton *et al.*, 2012).	● Bias in the individual-specific allele frequencies used to account for the different ancestries of admixed individuals can significantly impact relatedness estimates (Conomos *et al.*, 2016).<br><br>**ASSUMPTIONS**<br>NA.<br><br>**RUNNING TIME**<br>*REAP* took approximately 1 hr and 31 min to analyse 2,560 individuals and 100,000 SNPs. The REAP analysis of the HapMap MXL sample with 86 individuals and 150,872 SNPs took around 20 seconds (Intel Xeon quad-core 2.66 GHz processors with 16 GB of random-access memory) (Thornton *et al.*, 2012).
**PC-relate** (Conomos *et al.*, 2016).<br><br>**Citations (normalised):** 79 (26.33)	*PC-Relate* uses moment relatedness estimators to calculate the kinship coefficient, which uses the statistical method of moments to estimate the realised *k* coefficients; the proportion of genome at which two individuals share 0, 1, or 2 IBD genes (Wang, Sverdlov and Thompson, 2016).<br><br>*PC-Relate* uses principal components calculated to separate genetic correlations among sampled individuals into two separate components: a component for the allele sharing as a result of inherited IBD from recent common ancestors, which represents familial relatedness, and another component for allele sharing due to more distant common ancestry, which represents population structure (Conomos *et al.*, 2016).	**ADVANTAGES**<br>● Can work well with admixed samples (Conomos *et al.*, 2016).<br>● More accurate than REAP and RelateAdmix (Conomos *et al.*, 2016).<br>● Able to separate unrelated individuals from more distantly related ones (Moltke and Albrechtsen, 2014).<br><br>**LIMITATIONS**<br>• Bias in the individual-specific allele frequencies used to account for the different ancestries of admixed individuals can lead to significantly biased relatedness estimates (Conomos *et al.*, 2016).<br>• Likely suffers from the same limitation of PCA (Elhaik 2021).<br><br>**ASSUMPTIONS**<br>NA.<br><br>**RUNNING TIME**<br>*PC-Relate* analysed all 3,587 individuals in the WHI-SHARe Hispanic cohort with 87,180 SNPs in 12.1 minutes (Conomos *et al.*, 2016).
**PLINK** (Purcell *et al.*, 2007).<br><br>**Citations (normalised):** 16,675 (1389.58)	Methods for IBD detection identify similarities between haplotypes that are statistically unlikely to occur in the absence of IBD sharing (Durand, Eriksson and Mclean, 2014). *PLINK* incorporates a method of moments approach using an HMM to infer underlying IBD in chromosomal segments based on observed IBS states.	**ADVANTAGES**<br>● More accurate and has shown lower rates of false-positive results compared with *GERMLINE* (Purcell *et al.*, 2007).<br><br>**LIMITATIONS**<br>● Slower than GERMLINE (Gusev *et al.*, 2009).<br>● Has shown higher rates of false-positive results than *GERMLINE* (Gusev *et al.*, 2009).<br>● Produces high levels of false positives for second-degree relationships (Stevens *et al.*, 2011).

		**ASSUMPTIONS**<br>• Homogeneous, random-mating population (Purcell *et al.*, 2007).<br><br>**RUNNING TIME**<br>PLINK was able to analyse 2,000 individuals in 556 hours (2x2.4-GHz processors) (Gusev *et al.*, 2009).
**fastIBD**<br>(Browning and Browning, 2011).<br><br>**Citations (normalised):**<br>270 (33.75)	Methods for IBD detection identify similarities between haplotypes that are statistically unlikely to occur in the absence of IBD sharing (Durand, Eriksson and Mclean, 2014). *fastIBD* detects "seeds" of identical short haplotype matches and extends them to nearby sites.<br><br>The *fastIBD* algorithm samples a fixed number of haplotype pairs for each individual from the posterior haplotype distribution. Each sampled haplotype corresponds to a sequence of hidden Markov model (HMM) states. *fastIBD* searches for pairs of sampled haplotypes sharing the same sequence of HMM states for a set of consecutive markers. For each pair of individuals, overlapping shared haplotype tracts are merged and a score is calculated for each merged tract. If this score is below a user-specified threshold, the tract is printed to an output file (Gusev *et al.,* 2009).	**ADVANTAGES**<br>● Can be applied to large sample sizes across genome-wide SNP data (Browning and Browning, 2011).<br>● High power and low false-discovery rate compared to PLINK and GERMLINE (Browning and Browning, 2011).<br><br>**LIMITATIONS**<br>● Slower and less accurate than GERMLINE and PLINK (Gusev *et al.*, 2009; Browning and Browning, 2011).<br>● Requires phased data which is prone to phasing errors and increases computation time (Browning and Browning, 2011).<br>● Unfeasible to analyse over 100,000 individuals due to the phasing requirement which limits the number of individuals that can be phased at one time (Browning and Browning, 2011).<br><br>**ASSUMPTIONS**<br>NA.<br><br>**RUNNING TIME**<br>To analyse 4806 individuals and 37,645 SNPs, for which IBD was estimated for all possible pairs of individuals across the chromosome, the running time for *fastIBD* was approximately 17 hours (single core of an Intel Xeon E5620 Quad-Core compute node running at 2.40GHz) (Browning and Browning, 2011).

**GERMLINE**<br>(Gusev *et al.*, 2009).<br>**Citations (normalised):**<br>331 (33.10)	Methods for IBD detection identify similarities between haplotypes that are statistically unlikely to occur in the absence of IBD sharing (Durand, Eriksson and Mclean, 2014). *GERMLINE* detects “seeds” of identical short haplotype matches and extends them to nearby sites.<br><br>*GERMLINE* firstly detects completely identical match-seeds of potentially shared segments by creating a dictionary of allele combination words across the population observed at different slices along the genome. *GERMLINE* then uses a dynamic programming algorithm along different slices to extend these candidate matches and resolve the likelihood of IBD (Gusev *et al.*, 2009).	**ADVANTAGES**<br>● Faster than *fastIBD* and *PLINK* (Gusev *et al.*, 2009; Browning and Browning, 2011).<br>● More accurate than PLINK (Gusev *et al.*, 2009).<br>● Has shown lower rates of false-positive results than *PLINK* (Gusev *et al.*, 2009).<br>● Does not require phased data (Gusev *et al.*, 2009).<br><br>**LIMITATIONS**<br>• Has shown higher rates of false-positive results than *PLINK* (Browning and Browning, 2013).<br><br>**ASSUMPTIONS**<br>NA.<br><br>**RUNNING TIME**<br>GERMLINE was able to analyse 2,000 individuals in 40.3 hours compared to *PLINK*’s 556 hours (2x2.4-GHz processors) (Browning and Browning, 2013).

Figure S1: *The popularity (normalised number of citations) of the different GAI software, separated into model-based (red) and non-parametric (blue) tools. The tools were found using existing review papers and free search, using the search engines 'Google', 'Google Scholar' and the journal 'Bioinformatics' to search for keywords including: 'software', 'tools', 'inference', 'biogeographic', 'ancestry', 'kinship', and 'haplotype'. The number of citations for the paper proposing the tool, taken from 'Google Scholar', were compared for each tool within each domain. To account for the differences in the number of years since publication, the number of citations was normalised by dividing the number of citations by the number of years since publication.*

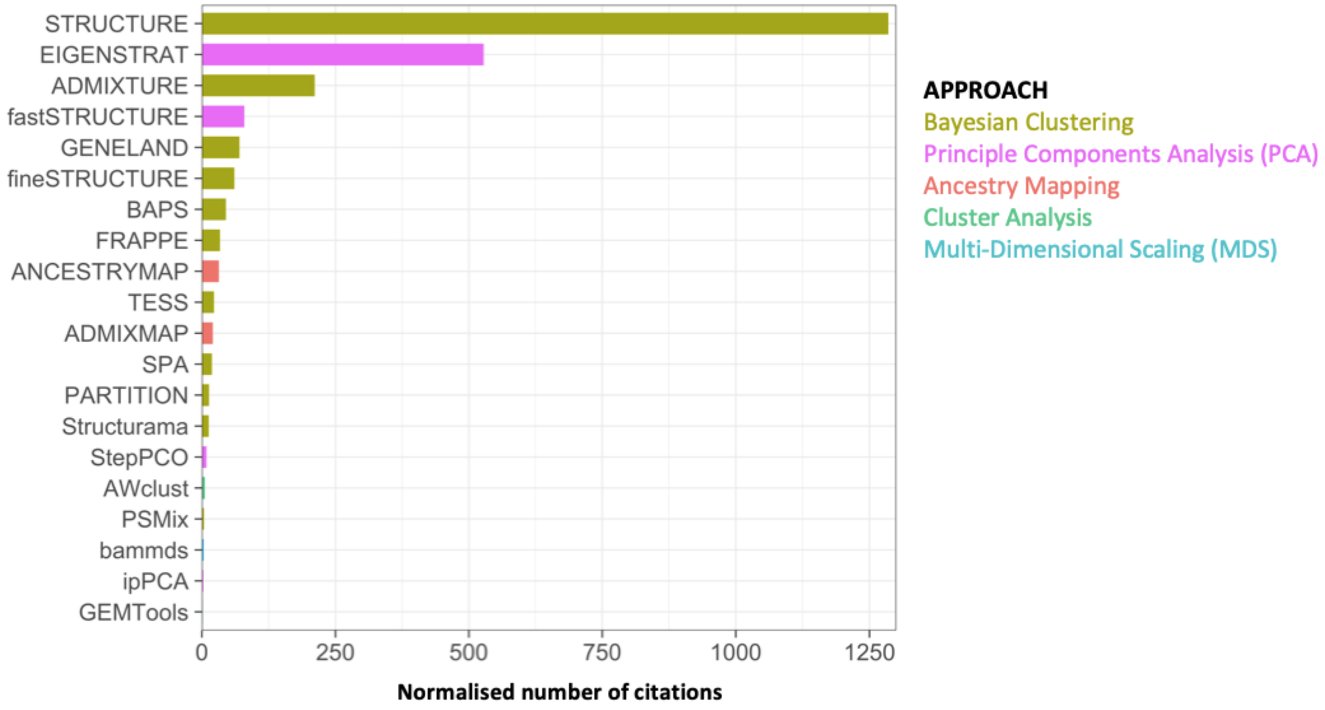

Figure S2: *The popularity (normalised number of citations) of the different LAI software, separated into their technologies: Hidden Markov Model (HMM) (green), Chromosome Painting (red) and Statistical Learning Algorithm (SLA) (blue). Finding the tools and calculating the normalised citation number was done as in Figure S1.*

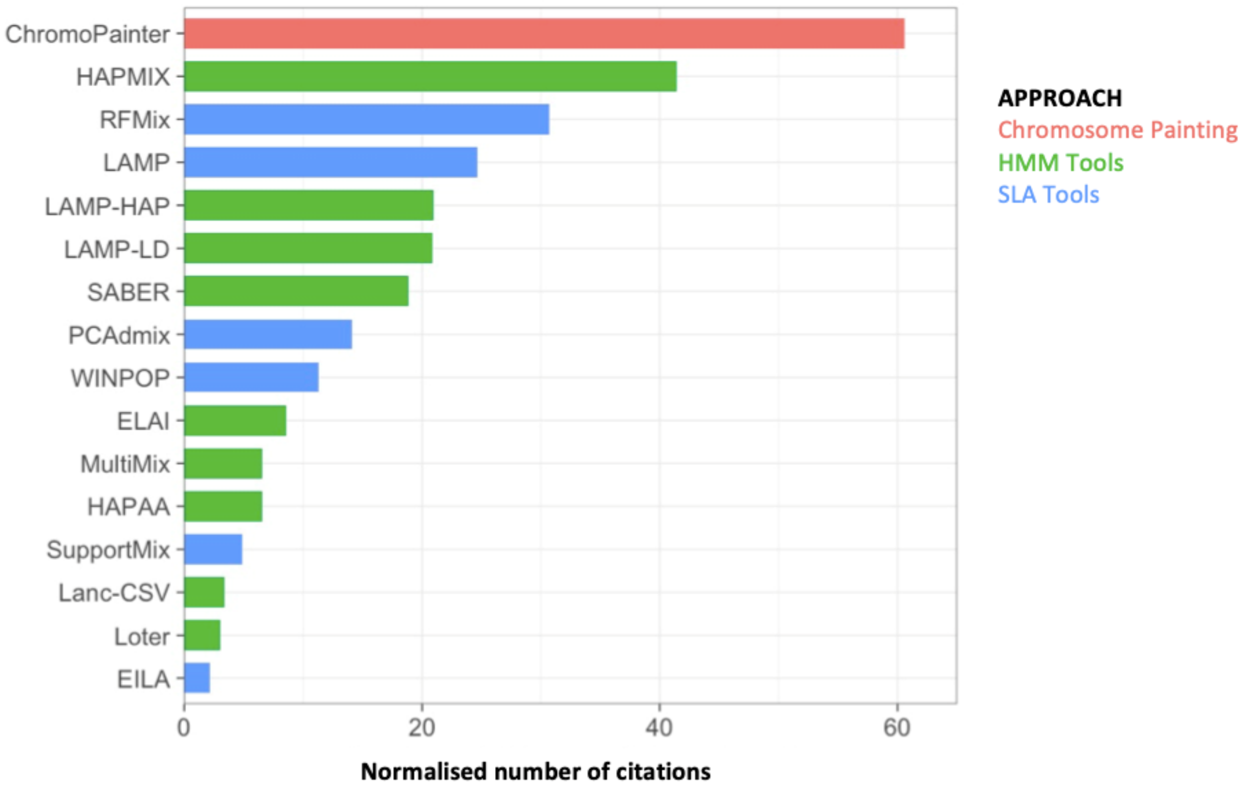

Figure S3: *The popularity (normalised number of citations) of the different kinship inference software, separated into their software strategies: Identity-By-Descent (IBD) detection (red) and Kinship Coefficient Estimation (blue). Finding the tools and calculating the normalised citation number was done as in Figure S1.*

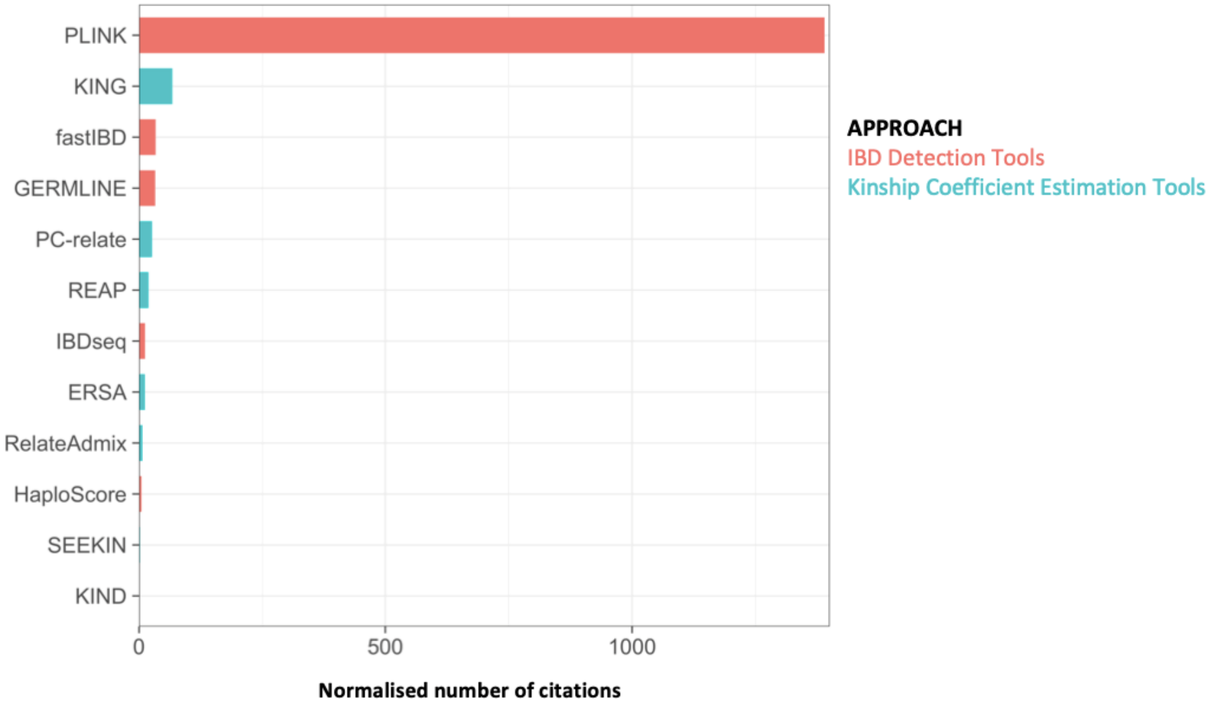

REFERENCES

Alexander, D. H. and Lange, K. (2011) 'Enhancements to the ADMIXTURE algorithm for individual ancestry estimation', *BMC Bioinformatics*, 12. doi: 10.1186/1471-2105-12-246.

Alexander, D. H., Novembre, J. and Lange, K. (2009) 'Fast model-based estimation of ancestry in unrelated individuals.', *Genome research*, 19(9), pp. 1655–1664. doi: 10.1101/gr.094052.109.

Baran, Y. *et al.* (2012) 'Fast and accurate inference of local ancestry in Latino populations', *Bioinformatics*, 28(10), pp. 1359–1367. doi: 10.1093/bioinformatics/bts144.

Basto, M. P. *et al.* (2016) 'Assessing genetic structure in common but ecologically distinct carnivores: The stone marten and red fox', *PLoS ONE*, 11(1). doi: 10.1371/journal.pone.0145165.

Brown, R. and Pasaniuc, B. (2014) 'Enhanced Methods for Local Ancestry Assignment in Sequenced Admixed Individuals', *PLoS Computational Biology*, 10(4). doi: 10.1371/journal.pcbi.1003555.

Browning, B. L. and Browning, S. R. (2011) 'A fast, powerful method for detecting identity by descent', *American Journal of Human Genetics*, 88(2), pp. 173–182. doi: 10.1016/j.ajhg.2011.01.010.

Browning, B. L. and Browning, S. R. (2013) 'Detecting identity by descent and estimating genotype error rates in sequence data', *American Journal of Human Genetics*, 93(5), pp. 840–851. doi: 10.1016/j.ajhg.2013.09.014.

Chen, C. *et al.* (2007) 'Bayesian clustering algorithms ascertaining spatial population structure: A new computer program and a comparison study', *Molecular Ecology Notes*, 7(5). doi: 10.1111/j.1471-8286.2007.01769.x.

Conomos, M. P. *et al.* (2016) 'Model-free Estimation of Recent Genetic Relatedness', *American Journal of Human Genetics*, 98(1), pp. 127–148. doi: 10.1016/j.ajhg.2015.11.022.

Corander, J., Waldmann, P. and Sillanpää, M. J. (2003) 'Bayesian analysis of genetic differentiation between populations.', *Genetics*, 163(1), pp. 367–374.

Dias-Alves, T., Mairal, J. and Blum, M. G. B. (2018) 'Loter: A software package to infer local ancestry for a wide range of species', *Molecular Biology and Evolution*, 35(9), pp. 2318–2326. doi: 10.1093/molbev/msy126.

Durand, E. *et al.* (2009) 'Spatial inference of admixture proportions and secondary contact zones', *Molecular Biology and Evolution*, 26(9), pp. 1963–1973. doi: 10.1093/molbev/msp106.

Durand, E. Y., Eriksson, N. and Mclean, C. Y. (2014) 'Reducing pervasive false-positive

identical-by-descent segments detected by large-scale pedigree analysis', *Molecular Biology and Evolution*, 31(8), pp. 2212–2222. doi: 10.1093/molbev/msu151.

Elhaik E. 2021. (2021) Why most Principal Component Analyses (PCA) in population genetic studies are wrong. bioRxiv.2021.2004.2011.439381. doi: 10.1101/2021.04.11.439381

Elhaik, E. and Ryan, D. M. (2019) 'Pair Matcher (PaM): fast model-based optimization of treatment/case-control matches', *Bioinformatics*, 35(13), pp. 2243–2250. doi: 10.1093/bioinformatics/bty946.

Falush, D., Stephens, M. and Pritchard, J. K. (2003) 'Inference of population structure using multilocus genotype data: linked loci and correlated allele frequencies.', *Genetics*, 164(4), pp. 1567–1587.

Guillot, G., Mortier, F. and Estoup, A. (2005) 'GENELAND: A computer package for landscape genetics', *Molecular Ecology Notes*, 5(2), pp. 712–715. doi: 10.1111/j.1471-8286.2005.01031.x.

Gusev, A. *et al.* (2009) 'Whole population, genome-wide mapping of hidden relatedness', *Genome Research*, 19, pp. 318–326. doi: 10.1101/gr.081398.108.

Intarapanich, A. *et al.* (2009) 'Iterative pruning PCA improves resolution of highly structured populations', *BMC Bioinformatics*, 10(382). doi: 10.1186/1471-2105-10-382.

Joseph, T. A. and Pe'er, I. (2018) 'Inference of Population Structure from Ancient DNA', *bioRxiv*, pp. 90–104. doi: 10.1007/978-3-319-89929-9_6.

Latch, E. K. *et al.* (2006) 'Relative performance of Bayesian clustering software for inferring population substructure and individual assignment at low levels of population differentiation', *Conservation Genetics*, 7, pp. 295–302. doi: 10.1007/s10592-005-9098-1.

Lawson, D. J. *et al.* (2012) 'Inference of population structure using dense haplotype data', *PLoS Genetics*, 8(1). doi: 10.1371/journal.pgen.1002453.

Leslie, S. *et al.* (2015) 'The fine-scale genetic structure of the British population', *Nature*, 519(7543), pp. 309–314. doi: 10.1038/nature14230.

Lombaert, E., Guillemaud, T. and Deleury, E. (2018) 'Biases of STRUCTURE software when exploring introduction routes of invasive species', *Heredity*, 120(6), pp. 485–499. doi: 10.1038/s41437-017-0042-1.

Manichaikul, A. *et al.* (2010) 'Robust relationship inference in genome-wide association studies', *Bioinformatics*, 26(22), pp. 2867–2873. doi: 10.1093/bioinformatics/btq559.

McVean, G. (2009) 'A genealogical interpretation of principal components analysis',

PLoS Genetics, 5(10). doi: 10.1371/journal.pgen.1000686.

Moltke, I. and Albrechtsen, A. (2014) 'RelateAdmix: A software tool for estimating relatedness between admixed individuals', *Bioinformatics*, 30(7), pp. 1027–1028. doi: 10.1093/bioinformatics/btt652.

Pan, X. *et al.* (2017) 'Fine population structure analysis method for genomes of many', *Scientific Reports*, 7(1). doi: 10.1038/s41598-017-12319-1.

Patterson, N. *et al.* (2004) 'Methods for High-Density Admixture Mapping of Disease Genes', *The American Journal of Human Genetics*, 74(5), pp. 979–1000. doi: 10.1086/420871.

Price, A. L. *et al.* (2006) 'Principal components analysis corrects for stratification in genome-wide association studies', *Nature Genetics*, 38, pp. 904–909. doi: 10.1038/ng1847.

Price, A. L. *et al.* (2009) 'Sensitive detection of chromosomal segments of distinct ancestry in admixed populations', *PLoS Genetics*, 5(6). doi: 10.1371/journal.pgen.1000519.

Pritchard, J. K., Stephens, M. and Donnelly, P. (2000) 'Inference of population structure using multilocus genotype data', *Genetics*, 55(2), pp. 945–959.

Purcell, S. *et al.* (2007) 'PLINK: a tool set for whole-genome association and population-based linkage analyses.', *American journal of human genetics*, 81(3), pp. 559–75. doi: 10.1086/519795.

Ramstetter, M. D. *et al.* (2017) 'Benchmarking relatedness inference methods with genome-wide data from thousands of relatives', *Genetics*, 207(1), pp. 75–82. doi: 10.1534/genetics.117.1122.

Sankararaman, S. *et al.* (2008) 'Estimating Local Ancestry in Admixed Populations', *American Journal of Human Genetics*, 82(2), pp. 290–303. doi: 10.1016/j.ajhg.2007.09.022.

Stevens, E. L. *et al.* (2011) 'Inference of relationships in population data using identity-by-descent and identity-by-state', *PLoS Genetics*, 7(9). doi: 10.1371/journal.pgen.1002287.

Tang, H. *et al.* (2005) 'Estimation of individual admixture: Analytical and study design considerations', *Genetic Epidemiology*, 28(4), pp. 289–301. doi: 10.1002/gepi.20064.

Thornton, T. *et al.* (2012) 'Estimating kinship in admixed populations', *American Journal of Human Genetics*, 91(1), pp. 122–138. doi: 10.1016/j.ajhg.2012.05.024.

Wang, B., Sverdlov, S. and Thompson, E. (2016) 'Efficient estimation of realized kinship from SNP genotypes', *Genetics*, 205(3), pp. 1–23. doi: 10.1534/genetics.116.197004.

Yuan, K. *et al.* (2017) 'Models, methods and tools for ancestry inference and admixture analysis', *Quantitative Biology*, 5(3), pp. 236–250. doi: 10.1007/s40484-017-0117-2.
